# Effectiveness and safety of exercise therapy in patients with myalgic encephalomyelitis/chronic fatigue syndrome: a meta-analysis

**DOI:** 10.3389/fneur.2025.1681990

**Published:** 2025-11-21

**Authors:** Zhenya Wei, Heying Wu, Chong Cui, Zixu Wang, Huazhong Xiong, Fujia Song, Jixiang Ren

**Affiliations:** 1 College of Chinese Medicine, Changchun University of Chinese Medicine, Changchun, Jilin, China; 2 Affiliated Hospital of Changchun University of Chinese Medicine, Changchun, Jilin, China

**Keywords:** chronic fatigue syndrome, exercise therapy, effectiveness, meta-analysis, Qigong

## Abstract

**Purpose:**

To promote the development of new therapies, we conduct a systematic review to evaluate the effectiveness and safety of exercise therapy for myalgic encephalomyelitis/chronic fatigue syndrome (ME/CFS).

**Methods:**

PubMed, Web of Science, Embase, and Cochrane were searched for exercise therapy studies on ME/CFS up to March 2024. The literature was updated on June 7, 2025. The meta-analysis was performed using Stata 17.0 and RevMan 5.4.

**Results:**

A total of 13 studies with 1,305 patients were analyzed. Exercise therapy improved overall scores on fatigue scale-14 [FS-14; MD = −0.48, 95% CI (−0.77, −0.19), *p* < 0.001] and reduced total fatigue score [MD = −1.59, 95% CI (−2.44, −0.75), *p* < 0.001]. With multidimensional fatigue inventory (MFI-20), it showed a non-significant reduction in general fatigue [MD = −0.23, 95% CI (−0.55, 0.10), *p* = 0.168]. Subgroup analysis showed that conventional exercise therapy mainly based on aerobic exercise was more effective in reducing total fatigue score [MD = −5.56, 95% CI (−8.74, −2.38), *p* = 0.001] than Qigong [MD = −0.09, 95% CI (−0.41, 0.23), *p* < 0.001]. However, Qigong was more effective in reducing mental fatigue [MD = −0.82, 95% CI (−1.38, −0.26), *p* = 0.004] compared with conventional exercise [MD = −3.40, 95% CI (−5.59, −1.21), *p* = 0.002].

**Conclusion:**

Evidence indicates that exercise therapy alleviates fatigue in ME/CFS patients, with varying effects across intervention types. Conventional aerobic exercise appears more effective for reducing overall fatigue than Qigong. However, Qigong shows greater benefits for reducing mental fatigue. Given the current limitations, the safety of exercise therapy requires further evaluation, and additional high-quality RCTs are warranted to validate these findings.

## 1 Introduction

Myalgic encephalomyelitis/chronic fatigue syndrome (ME/CFS) is a chronic disease primarily characterized by unexplained, persistent fatigue. It is often accompanied by multisystem symptoms, such as unrefreshing sleep, cognitive dysfunction, orthostatic intolerance, and pain (1). Since the outbreak of COVID-19 in 2019, ME/CFS has been recognized as one of the most common clinical subtypes of long COVID. It is estimated that nearly half of patients with long-term post-COVID symptoms meet the diagnostic criteria for ME/CFS, which has garnered increasing global attention (2). The hallmark symptom of ME/CFS is a delayed, worsening systemic response to even minimal exertion, which is not relieved by rest. This disabling and often permanent condition severely restricts patients' daily functioning, resulting in a lower quality of life than many other chronic diseases and imposing profound social, educational, and economic burdens (3). Currently, the etiology of ME/CFS remains unclear, and its diagnosis relies mainly on clinical manifestations and exclusion of other diseases, thus posing significant therapeutic challenges. Existing pharmacological treatments are largely symptomatic, targeting the patient's main complaints. However, a complete cure is rarely achievable, and most patients present in the middle or late stages of the disease (4). Consequently, non-pharmacological interventions that are easy to implement, such as exercise therapy, play an especially important role in ME/CFS management.

Exercise therapy, as a key component of non-pharmacological management for ME/CFS, encompasses various forms, including graded exercise therapy (GET) that emphasizes progressive increases in activity, moderate-intensity aerobic exercise aimed at improving cardiopulmonary function, and mind-body practices derived from traditional Chinese exercises, such as Qigong (5). Although some randomized controlled trials (RCTs) have reported beneficial effects of exercise therapy on reducing fatigue, improving physical function, and quality of life, significant methodological heterogeneity and inconsistent findings have also been noted. Zhao et al. (6) found that progressive aerobic exercise significantly alleviated fatigue in ME/CFS patients. In contrast, Gaunt et al. (7) indicated that GET had a limited effect on reducing fatigue severity and physical fatigue in ME/CFS patients. Qigong is a traditional Chinese exercise system that includes practices such as Tai Chi and Baduanjin. With the development of traditional Chinese medicine, Qigong and related mind-body exercises have become widely used for managing fatigue, depression, and other conditions. These practices emphasize the integration of mind and body and the harmonization of qi and blood to improve energy flow, thereby regulating core symptoms of ME/CFS. Their gentle, low-intensity nature makes them particularly suitable for ME/CFS patients who are sensitive to exercise intensity or have more severe disease (8). Currently, the efficacy and risks of different exercise therapies have not been systematically compared.

The present meta-analysis aimed to evaluate the effects of different exercise therapies, including GET, aerobic exercise, and Qigong, on patients with ME/CFS. Our findings may provide a comprehensive understanding of their impact on ME/CFS and offer evidence-based guidance for the clinical management of ME/CFS.

## 2 Methods

This study was conducted following the Preferred Reporting Items for Systematic Reviews and Meta-Analyses. The study protocol was registered in the International Prospective Register of Systematic Reviews (registration No.: CRD42024524753). This study adhered to all PRISMA guidelines and reported the required information accordingly.

### 2.1 Literature search

PubMed, Web of Science, Embase, and Cochrane were searched for relevant studies on exercise therapy in ME/CFS patients. The search period spanned from database inception to March 2024. An updated search was conducted on June 7, 2025, to include the latest studies. The search used exercise therapy and ME/CFS as both subject terms and free words, including “Chronic Fatigue Syndromes,” “Fatigue Syndrome, Chronic,” “Exercise Therapy,” and “exercise treatment.” The specific search strategies are illustrated in Supplementary Attachments 1–8.

### 2.2 Inclusion criteria

i) Subjects: patients diagnosed with ME/CFS;
ii) Intervention measures: exercise therapy, including GET, graded exercise self-help (GES), gradual exercise therapy, Qigong therapy (such as Qigong, Baduanjin, Wu Xing Ping Heng Gong, Yijinjing, and Prolong Life with Nine Turn Method), and graded exercise with pacing;
iii) Control measures: non-intervention measures, CBT, specialist medical care, adaptive pacing therapy, standard medical care, and heart rate variability biofeedback therapy;
iv) Outcome measures: at least one fatigue measure was reported, such as fatigue scale-14 (FS-14/Chalder fatigue questionnaire) (9) multidimensional fatigue inventory (MFI-20) (10), and adverse effects;
v) Study type: RCT.

### 2.3 Exclusion criteria

i) No available full text;
ii) Unable to extract valid data;
iii) Duplicates;
iv) Non-English publications;
v) Non-clinical controlled studies, such as animal experiments, case reports, letters, and reviews;
vi) Intervention measures not aligned;
vii) Presence of comorbidities.

### 2.4 Study selection

Two investigators independently screened the studies for inclusion. According to the predefined inclusion criteria, all relevant studies were downloaded and imported into EndNote 21. After removing duplicates, the titles and abstracts of the remaining studies were reviewed to exclude those that did not meet the criteria. Finally, full texts of the remaining studies were examined to further exclude any non-compliant studies and to determine the final studies that met the inclusion criteria.

### 2.5 Data extraction

Two investigators independently extracted information from each included study, encompassing the title, authors, country, study type, age, and intervention measures, along with participant numbers, intervention duration, and control measures accompanied by participant numbers. Any discrepancies were resolved through discussions with a third investigator.

### 2.6 Risk of bias and quality assessment

Risk of bias in the included studies was assessed by two investigators utilizing the Cochrane risk of bias tool (11). The assessment encompassed the following entries: (i) random sequence generation; (ii) allocation concealment; (iii) blinding of both investigators and participants; (iv) blinding for outcome assessment; (v) completeness of outcome data; (vi) selective reporting of results; (vii) other sources of bias. Each entry was assessed as low risk, high risk, or unclear risk. The Jadad scale (12) was leveraged to assess the quality of the included studies from the following entries: (i) random sequence generation; (ii) concealment of the random sequence; (iii) blinding; (iv) withdrawals and dropouts. Each entry was rated as 0–2 points. A score between 4 and 7 indicated high quality, while a score ranging from 0 to 3 signified low quality.

### 2.7 Data analysis

Stata 17.0 and Reveman 5.4 were applied to perform data analysis. The primary outcome measures in this study included FS-14, MFI-20, and adverse effects. Among them, FS-14 and MFI-20 were continuous variables, and the standardized mean difference (SMD) with a 95% confidence interval (CI) was utilized as the effect size. Adverse effects were binary variables, and relative risk (RR) with a 95% CI was used as the effect size. Heterogeneity was represented by the *I*^2^ statistic. When *I*^2^ > 50% or *p* < 0.05, it signified a high level of heterogeneity, and a random-effects model would be utilized. Conversely, when *I*^2^ ≤ 50% and *p* > 0.05, a fixed-effects model was appropriate. When the number of studies included exceeded four, a risk of bias and sensitivity analysis would be conducted. If more than 10 studies were included, Egger's test would be employed for risk of bias assessment. If 10 or fewer studies were included, a funnel plot would be utilized to evaluate publication bias. All data analyses were conducted utilizing a two-tailed approach. A *p*-value below 0.05 was deemed statistically significant.

## 3 Results

### 3.1 Study selection

**Figure 1:**
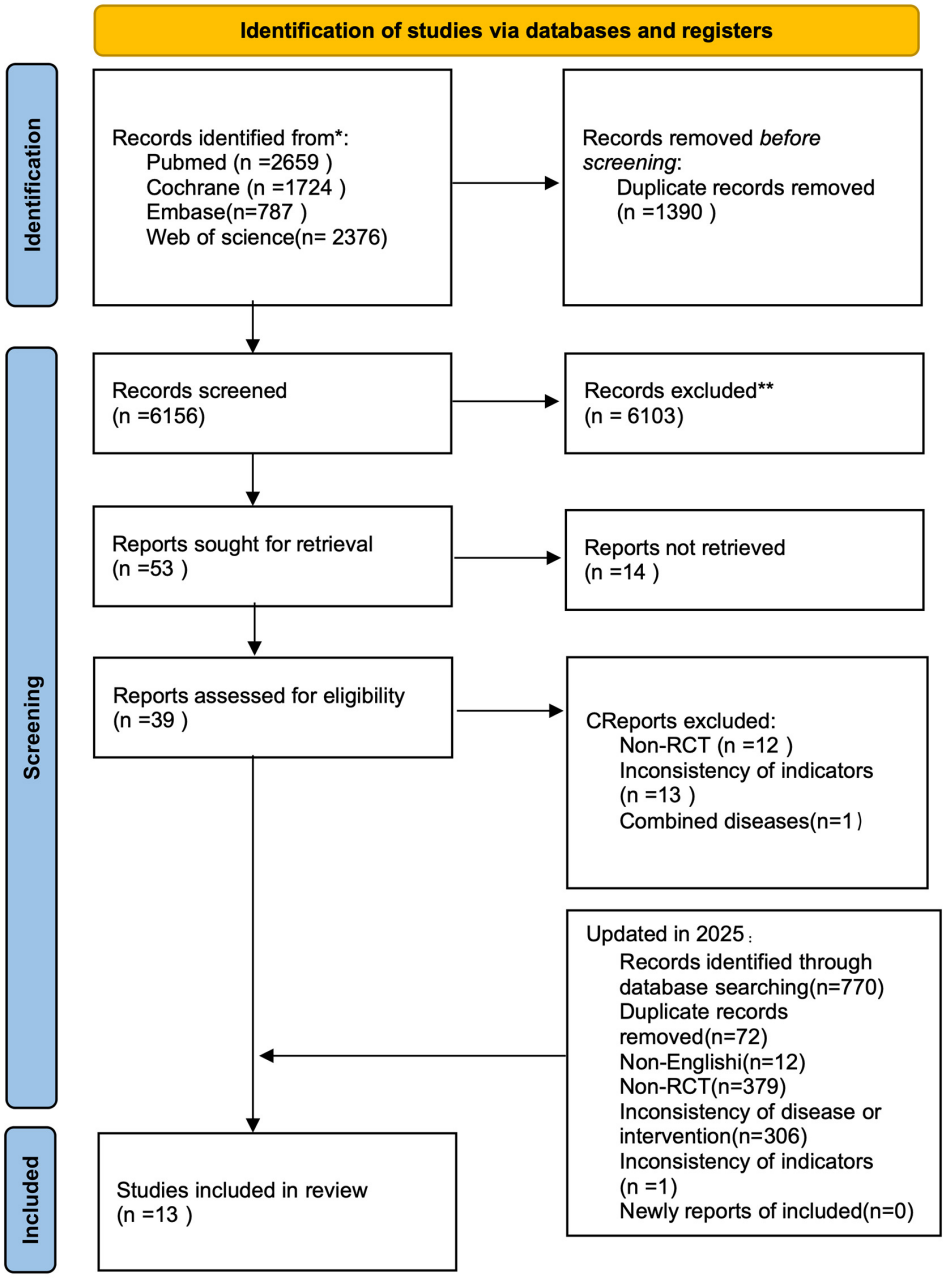
Flowchart of the study screening process.

In total, 7,546 studies were retrieved from four databases: PubMed (2,659), Cochrane (1,724), Embase (787), and Web of Science (2,376). After removing 1,390 duplicates, 6,103 articles were further excluded based on title and abstract screening. The remaining 53 studies underwent full-text review, and 13 RCTs were subsequently excluded (6, 13–24). The literature search was updated on June 7, 2025. A total of 770 studies were retrieved from PubMed (219), Cochrane (203), Embase (119), and Web of Science (229). No additional studies were eligible for inclusion after screening. The study screening process is illustrated in Figure 1.

### 3.2 Basic characteristics of the included studies

This study included 1,305 patients, with 659 in the intervention group and 646 in the control group. The number of patients in the intervention groups across different studies varied, ranging from 11 to 160 individuals. Windthorst et al. (22) involved the fewest participants, with a total of 26 individuals, while White et al. (14) engaged the largest number of participants, totaling 319. Eight studies (6, 15–18, 21, 23, 24) were conducted in China, two (13, 14) in the United Kingdom, one (20) in New Zealand, one (19) in Australia, and one (22) in Germany. There were five exercise therapies involved in total. Two studies (6, 19) involved exercise interventions, one (13) involved GES therapy, two (14, 22) involved GET, one (21) focused on gradual exercise therapy, one (20) involved graded exercise with pacing, and six (15–18, 23, 24) examined Qigong therapy. There were three primary outcome measures. Ten studies (6, 13–21) involved FS-14, three (22–24) focused on MFI-20, and two (13, 14) addressed adverse effects. The basic characteristics of the included studies are presented in Table 1.

**Table 1:** Basic characteristics of the included studies.

**Study**	**Country**	**Type**	**Patients**	**Age ($\bar{\text{x}}$ ±s, I/C)**	**Gender (f/m)**	**Intervention**	***N***	**Control**	***N***	**Intervention duration**	**Outcome**	**Jaded**

			**Name**			**Name**		**Name**				
Shanguang Zhao (2022)	China	RCT	ME/CFS	16.57 ± 0.28/16.72 ± 0.41	–	Aerobic training	23	Control	23	12 weeks	a (a1, a2, a3)	2
Lucy V. Clark (2017)	UK	RCT	ME/CFS	38.1 ± 11.1/38.7 ± 12.7	167/44	Guided graded exercise self-help	107	SMC	104	12 weeks	a (a4), c, d	5
PD White (2011)	UK	RCT	ME/CFS	39 ± 12/39 ± 11	244/75	Graded exercise therapy	160	Adaptive pacing therapy	159	24 weeks	a (a4), c, d, e	5
Rona Moss-Morris (2005)	New Zealand	RCT	ME/CFS	36.72 ± 11.83/45.48 ± 10.45	36/13	Graded exercise therapy	25	Standard medical care	24	12 weeks	a (a1, a2, a3)	5
Haibo Xu (2021)	China	RCT	ME/CFS	20.44 ± 5.17/–	48/22	Gradual exercise	35	Control	35	6 months	a (a1, a2, a3)	2
Jie Li (2015)	China	RCT	ME/CFS	46 ± 7.25/45 ± 4.75	40/6	Qigong (wu xing ping heng gong)	22	Control	24	3 months	a (a1, a2, a3)	4
Jessie S.M. Chan (2013)	China (Hong Kong)	RCT	ME/CFS	42.4 ± 6.7/42.5 ± 6.4	105/32	Qigong (wu xing ping heng gong)	72	Control	65	4 months	a (a1, a2, a3)	4
Jessie S.M. Chan (2014)	China (Hong Kong)	RCT	ME/CFS	39.1 ± 7.8/38.9 ± 8.1	108/42	Qigong (Baduanjin)	75	Control	75	9 weeks	a (a1, a2, a3)	4
Rainbow T.H (2012)	China (Hong Kong)	RCT	ME/CFS	42.1 ± 7.3/42.5 ± 5.5	51/13	Qigong (wu xing ping heng gong)	33	Control	31	4 months	a (a1, a2, a3)	4
Fangfang Xie (2023)	China	RCT	ME/CFS	35.13 ± 6.15/36.70 ± 8.47	27/12	Qigong (Yijinjing)	19	CBT	20	12 weeks	b (b1, b2, b3)	5
Fangfang Xie (2022)	China	RCT	ME/CFS	37.943 ± 11.344/37.343 ± 9.864	53/36	Qigong (PLNTM)	45	CBT	44	12 weeks	b (b1, b2, b3)	5
Karen E Wallman (2004)	Australia	RCT	ME/CFS	45 ± 14.5	–	Graded exercise therapy	32	Control	29	12 weeks	a (a1, a2, a3)	3
Petra Windthorst (2016)	Germany	RCT	ME/CFS	50.0 ± 10.9/51.4 ± 8.1	–	Graded exercise therapy	11	HRV-BF	13	8 weeks	b (b1, b2, b3)	3

CBT, cognitive behavioral therapy; Qigong (PLNTM), Qigong (prolong life with nine turn method); HRV-BF, heart rate variability biofeedback therapy.

a, fatigue scale-14, (FS-14/Chalder fatigue questionnaire; a1, Total fatigue score; a2, Physical fatigue score; a3, Mental fatigue score, a4, cFQ); b, MFI-20, The Multidimensional Fatigue Inventory (c1, General fatigue; c2, Physical fatigue; c3, Mental fatigue); c, Non-serious adverse events; d, Serious adverse events; e, Serious adverse reaction.

### 3.3 Risk of bias and quality assessment

**Figure 2:**
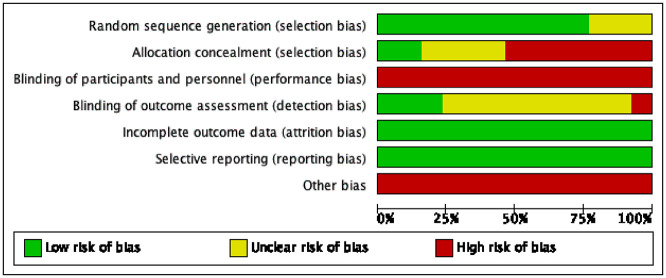
Table for risk of bias assessment.

**Figure 3:**
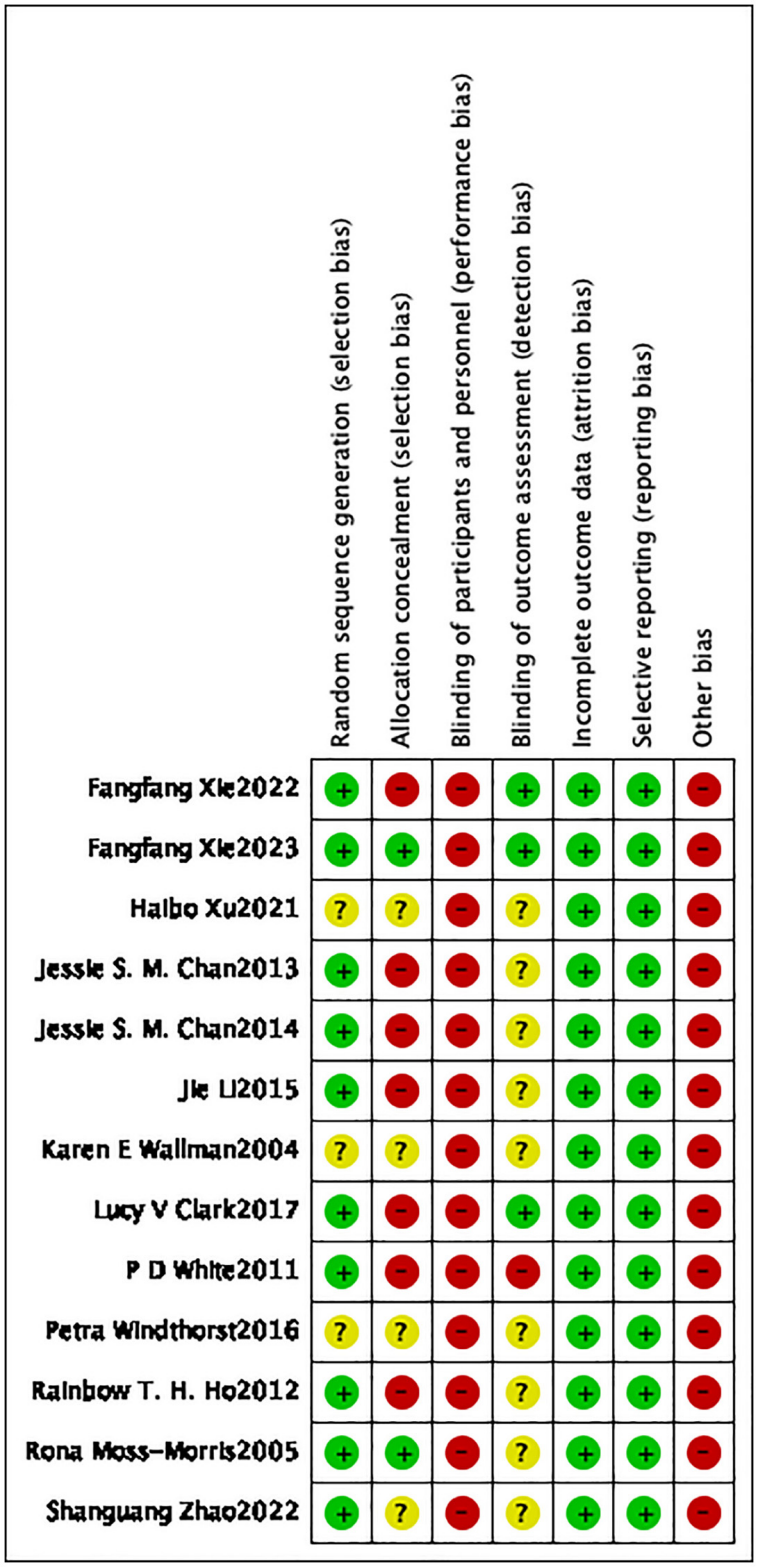
Summary chart of risk of bias.

Risk of bias in the included studies was assessed by means of the Cochrane risk-of-bias tool (10). The results are depicted in Figures 2, 3. Ten studies (6, 13–19, 23, 24) explicitly stated that the random sequences were produced by a computer and were judged to be low risk. In contrast, three studies (20–22) did not provide specific details regarding this aspect and were classified as having an unclear risk. Regarding allocation concealment, six studies (13–18) directly informed patients of the method of participation and were judged to be high risk. Three studies (19, 23, 24) were judged to be low risk due to the use of sealed opaque envelopes. Moreover, four other studies (6, 20–22) were deemed to have an unclear risk because they did not detail whether there was allocation concealment. Due to the unique nature of exercise therapy, it was challenging to implement blinding in intervention measures for patients. Consequently, all 13 studies were assessed as having a high risk of bias. Regarding blinding for outcome assessment, three studies (13, 23, 24) were judged to be at low risk, and nine studies (6, 15–22) did not clearly mention blinding and were rated as unclear, and one study (14) had evaluators who were aware of the interventions and was rated as high risk. The data from all 13 studies were complete, and no biases related to selective reporting of results were detected. Therefore, both follow-up bias and reporting bias were assessed as low risk. Regarding other potential sources of bias, eight studies (15–18, 20, 22–24) recruited participants through media, posters, and local communities. This recruitment method may introduce self-selection bias, thereby limiting the representativeness of the sample and reducing the generalizability of the findings to the broader ME/CFS population. Five studies (6, 13, 14, 19, 21) recruited participants from specific settings. For example, pdwhite (14) and lucyvclark (13) enrolled participants from ME/CFS specialty clinics, Xu (21) recruited patients diagnosed and treated in hospitals, and SZ2022 (6) recruited 46 diagnosed male high school students from a single school. These sampling approaches may introduce selection bias related to healthcare-seeking behavior or disease severity. Moreover, due to study design, all 13 included studies assessed outcomes using subjective tools such as the Chalder Fatigue Scale, without incorporating objective indicators. This may raise concerns about measurement bias. Four studies (17, 19, 20, 24) had sample sizes below 50 participants, which may further increase the risk of bias. Therefore, all 13 studies were judged to be at high risk for other biases. The Jaded scale was employed to assess the quality of the included studies (12). The results are illustrated in Table 1. Among random sequence generation and allocation concealment, nine studies (13–19, 23, 24) were operated by computer, receiving a score of 2 points. Additionally, four studies (6, 20–22) were randomly assigned but did not specify the exact methods used, resulting in a score of 1 point. In terms of blinding, due to the specific nature of exercise therapy, none of the 13 studies were blinded and scored 0 points. Seven studies (13, 14, 19, 20, 22–24) described reasons for patient withdrawal and dropout, receiving a score of 1, while six studies (6, 15–18, 21) did not provide detailed descriptions, receiving a score of 0. In summary, five studies (13, 14, 19, 23, 24) received a score of 5, while four studies (15–18) scored 4. Consequently, nine studies were classified as high quality in total. Two studies (20, 22) received a score of 3, while two other studies (6, 21) were assigned a score of 2. In total, four studies were classified as low quality.

### 3.4 Meta-analysis

The present study focused on two primary outcome measures, namely FS-14 and MFI-20. FS-14 was categorized into three assessment dimensions: total fatigue, physical fatigue, and mental fatigue. MFI-20, in addition to these three dimensions, also reported the assessment results for two additional dimensions: reduced activity and reduced motivation. Two studies reported adverse effects (13, 14) categorized into three types: non-serious adverse events, serious adverse events, and serious adverse reactions.

**Figure 4:**
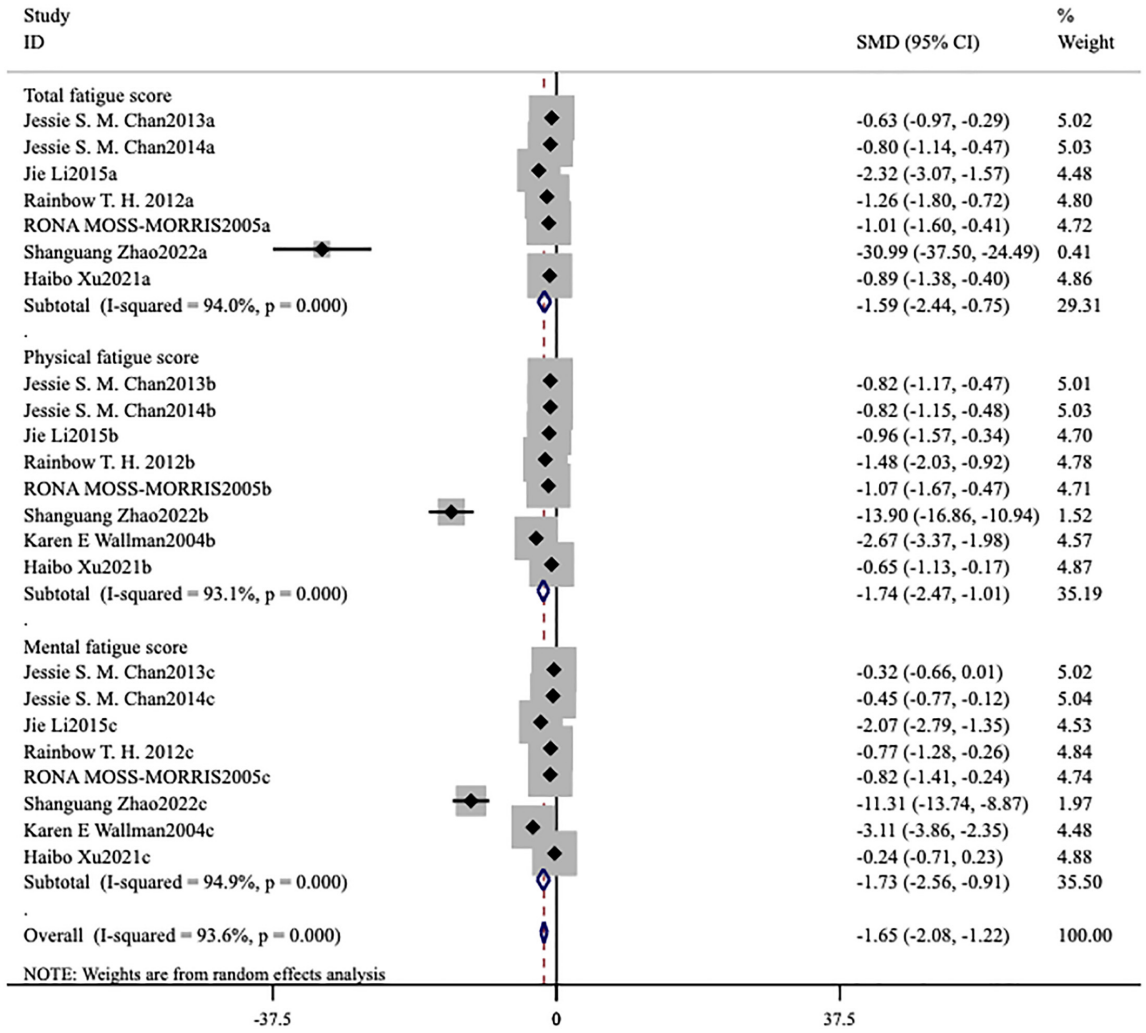
Meta-analysis of exercise therapy for chronic fatigue syndrome on fatigue scale-14 (three indicators).

**Figure 5:**
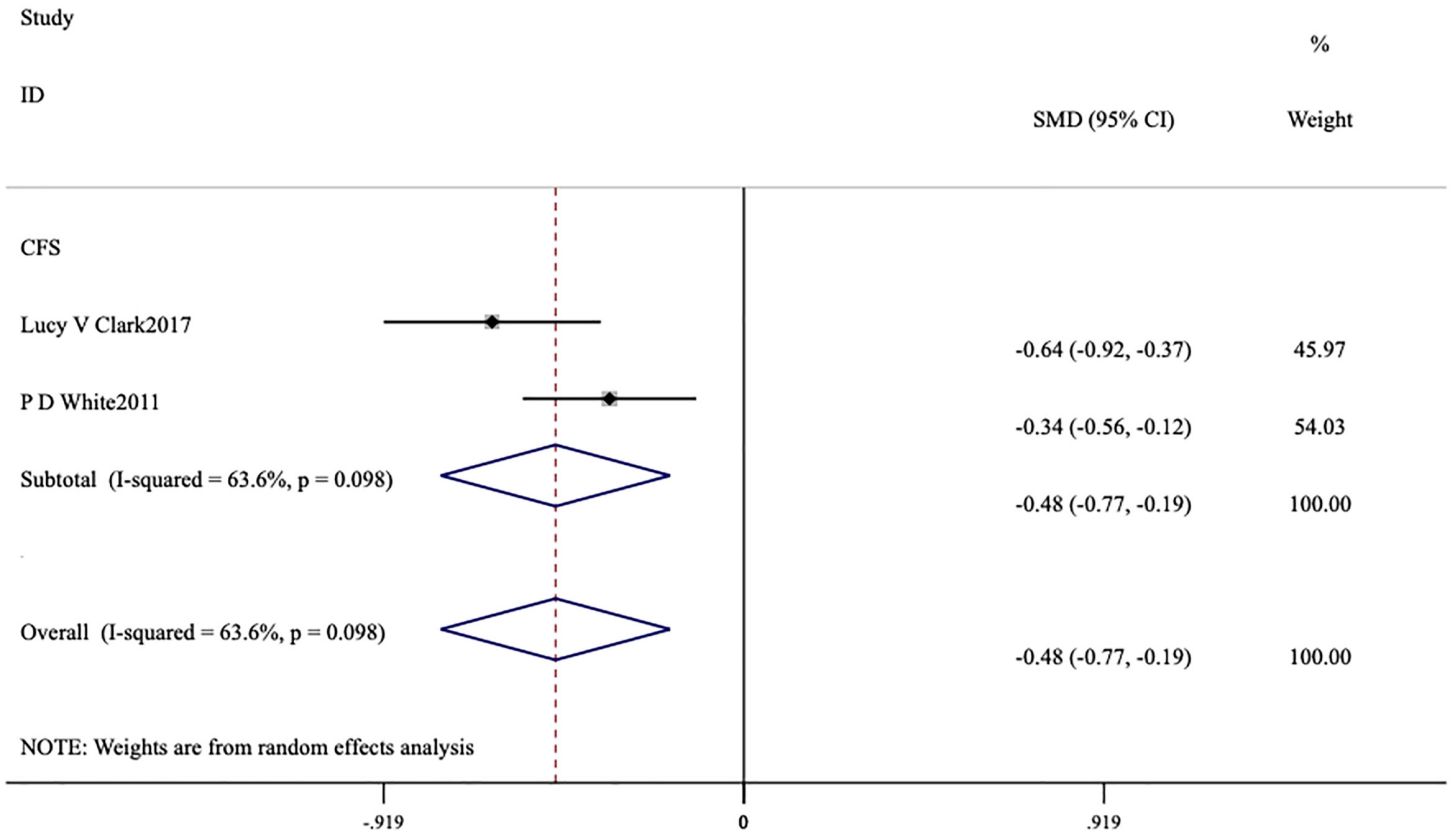
Meta-analysis of exercise therapy for chronic fatigue syndrome on fatigue scale-14 (total index).

**Figure 6:**
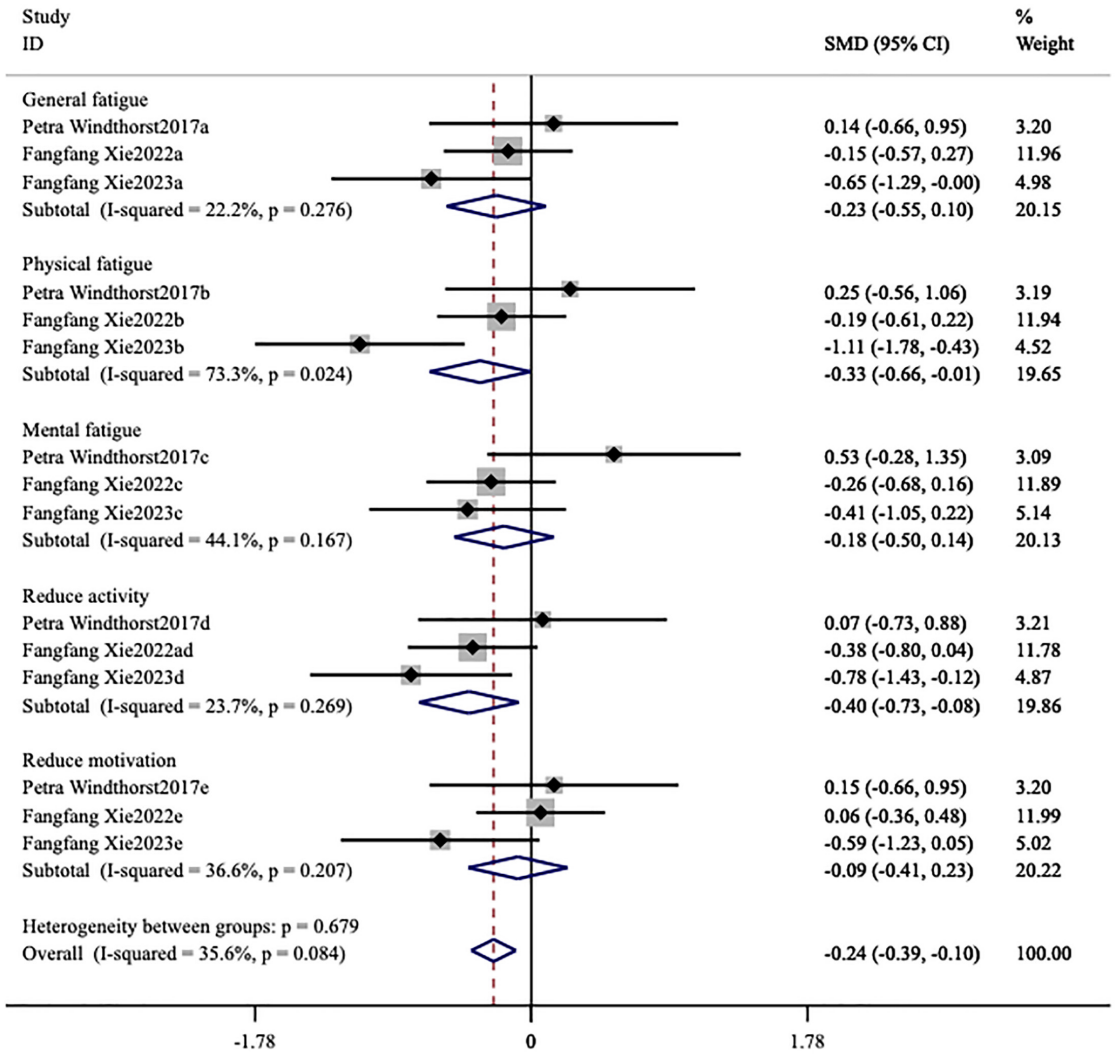
Meta-analysis of exercise therapy for chronic fatigue syndrome on multidimensional fatigue inventory.

The forest plot demonstrated that the heterogeneity among the studies for FS-14 (*I*^2^ = 93.5%, *p* < 0.001) was significant (Figures 4, 5), and a random-effects model was utilized. The heterogeneity among the studies for MFI-20 (*I*^2^ = 35.6%, *p* = 0.084) was relatively low (Figure 6), and a fixed-effects model was leveraged.

#### 3.4.1 FS-14

Ten studies (6, 13–21) assessed this fatigue scale. The forest plot demonstrated that the heterogeneity among the studies for FS-14 (*I*^2^ = 93.5%, *p* < 0.001) was significant (Figures 4, 5), and a random-effects model was therefore applied. The meta-analysis showed that in seven studies (6, 15–19, 21) patients in the intervention group had lower total fatigue compared to those in the control group [MD = −1.59, 95% CI (−2.44, −0.75), *p* < 0.001; Figure 4]. Eight studies (6, 15–21) reported changes in both physical fatigue and mental fatigue. Compared to the control group, exercise therapy notably alleviated physical fatigue [MD = −1.74, 95% CI (−2.47, −1.01), *p* < 0.001] and mental fatigue [MD = −1.73, 95% CI (−2.56, −0.91), *p* < 0.001] for patients. The overall score on the scale was utilized to compare the treatment effects before and after intervention. It was found that the overall score of ME/CFS in the intervention group showed a remarkable decrease [MD = −0.48, 95% CI (−0.77, −0.19), *p* < 0.001; Figure 5].

#### 3.4.2 MFI-20

The MFI-20 comprised five distinct dimensions (10) (Figure 6). Three studies (22–24) used this fatigue scale as the primary outcome measure. The heterogeneity among the studies for MFI-20 (*I*^2^ = 35.6%, *p* = 0.084) was relatively low (Figure 6), and a fixed-effects model was therefore used. Compared to the control group, Physical fatigue [MD = −0.33, 95% CI (−0.66, −0.01), *p* = 0.046] and reduced activity [MD = −0.40, 95% CI (−0.73, −0.08), *p* = 0.001] notably improved. No statistically significant effects were observed for general fatigue [MD = −0.23, 95% CI (−0.55, 0.10), *p* = 0.168], mental fatigue [MD = −0.18, 95% CI (−0.50, 0.14), *p* = 0.281], and reduced motivation [MD = −0.09, 95% CI (−0.41, 0.23), *p* = 0.593].

#### 3.4.3 Subgroup analysis of the exercise group and Qigong group

**Figure 7:**
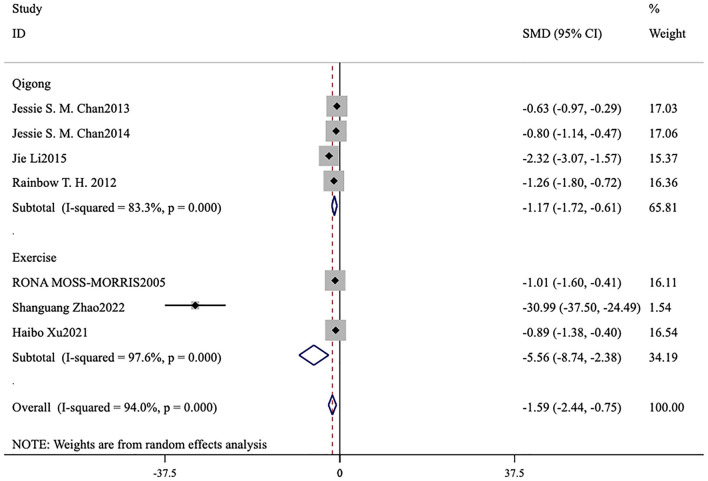
Meta-analysis of conventional exercise therapy and Qigong for total fatigue on fatigue scale-14.

**Figure 8:**
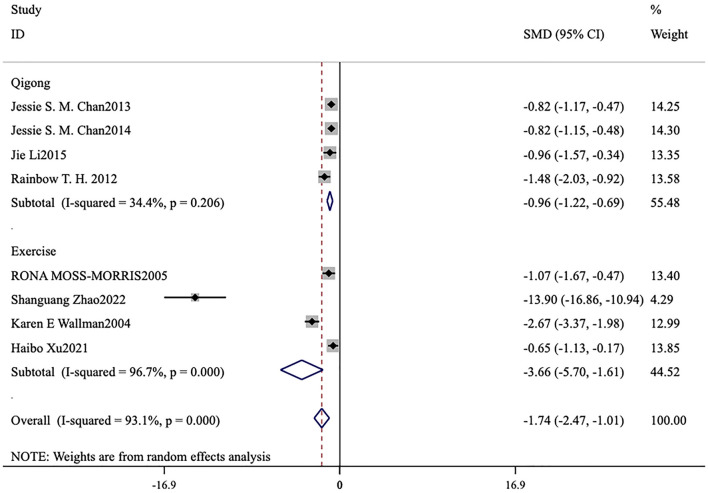
Meta-analysis of conventional exercise therapy and Qigong for physical fatigue on fatigue scale-14.

**Figure 9:**
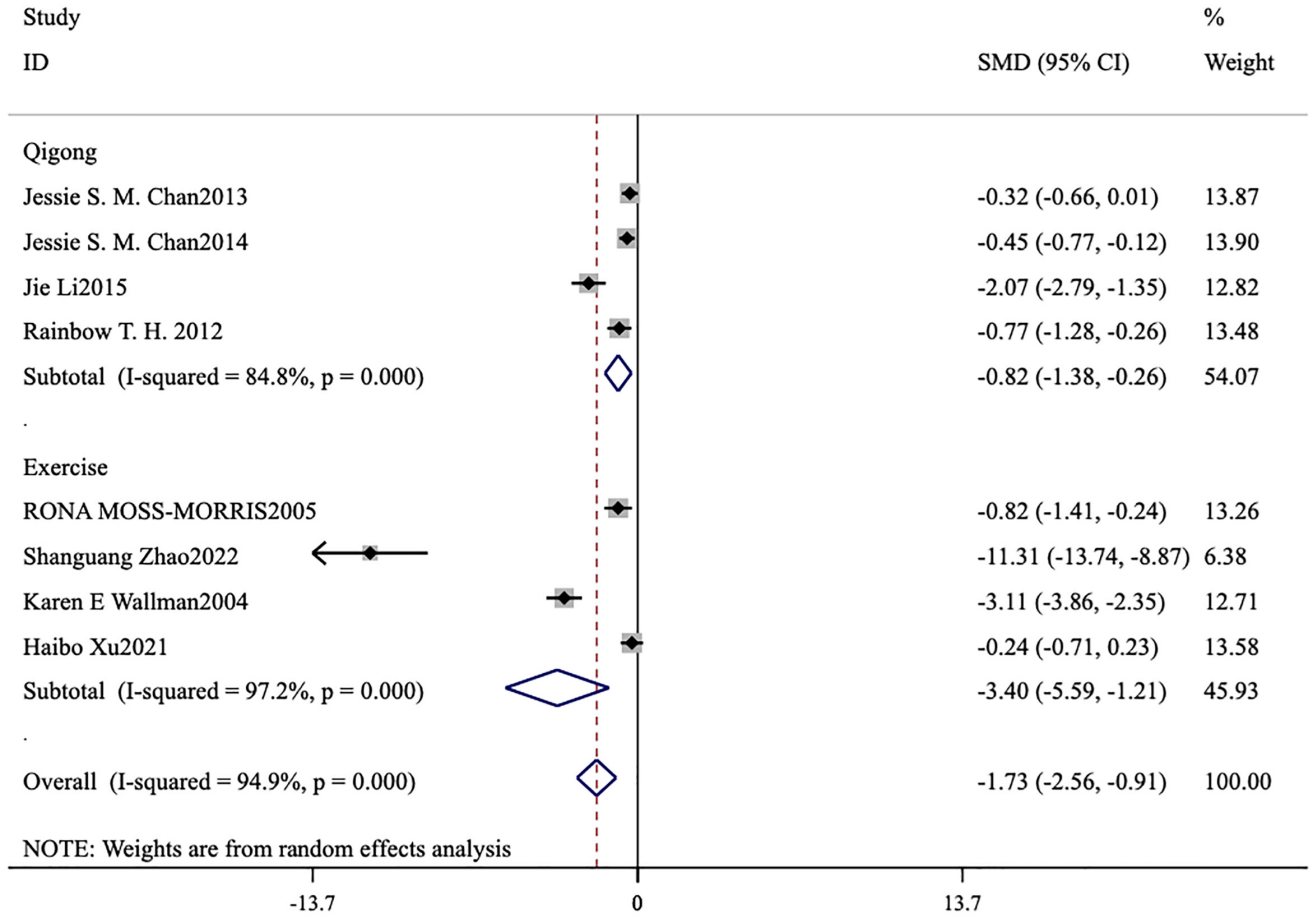
Meta-analysis of conventional exercise therapy and Qigong for mental fatigue on fatigue scale-14.

Under the premise of using FS-14 as the primary outcome measure, a meta-analysis was conducted by categorizing the intervention measures into two groups: a conventional exercise therapy group focused on aerobic exercise and a Qigong group. The forest plots reveal that, for reducing total fatigue, conventional exercise therapy [MD = −5.56, 95% CI (−8.74, −2.38), *p* = 0.001] outperformed Qigong [MD = −0.09, 95% CI (−0.41, 0.23), *p* < 0.001; Figure 7]. For reducing physical fatigue, conventional exercise therapy [MD = −3.66, 95% CI (−5.70, −1.61), *p* < 0.001] was also more effective than Qigong [MD = −0.96, 95% CI (−1.22, −0.69), *p* < 0.001; Figure 8]. In terms of improving mental fatigue, Qigong [MD = −0.82, 95% CI (−1.38, −0.26), *p* = 0.004] outperformed conventional exercise therapy [MD = −3.40, 95% CI (−5.59, −1.21), *p* = 0.002; Figure 9].

#### 3.4.4 Adverse effects

Two studies (13, 14) reported adverse effects. The safety outcomes were non-serious adverse events, serious adverse events, severe adverse reactions to the intervention, severe deterioration, and voluntary withdrawal. In one study (13) by Clark et al., ~1/4 of participants in both the intervention group and the control group reported non-serious adverse events, with no obvious difference between the two groups. Both groups reported one case of serious adverse events, and neither group reported any serious adverse reactions. The difference in severe deterioration between the two groups was not evident. Due to the insufficient occurrence of serious adverse events, their study did not compare the safety of interventions. In another study (14), non-serious adverse events were frequently observed, while serious adverse events and severe adverse reactions were relatively rare. Instances of severe deterioration were uncommon.

### 3.5 Publication bias and sensitivity analysis

**Figure 10:**
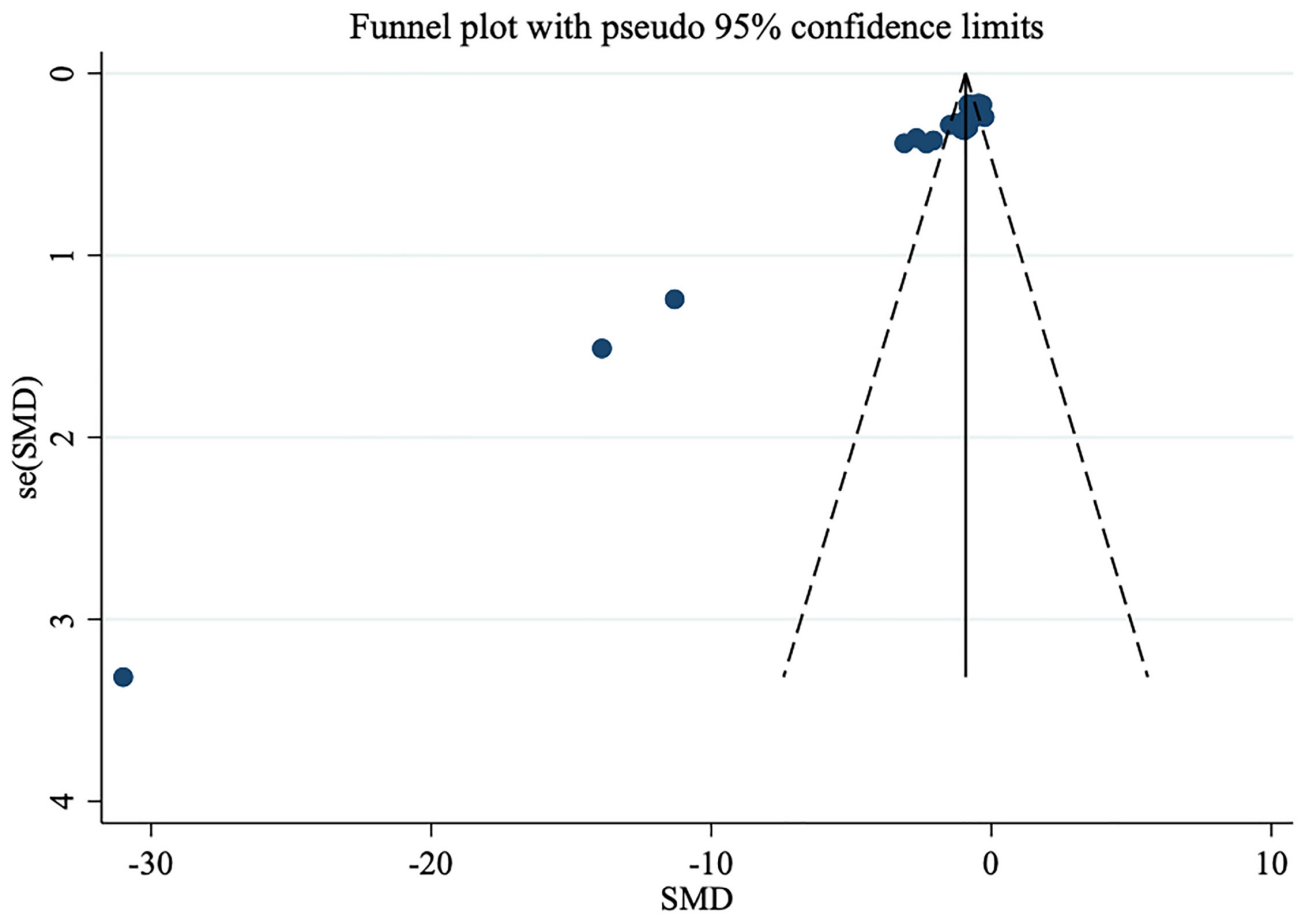
Funnel plot for publication bias in studies on exercise therapy for chronic fatigue syndrome.

**Figure 11:**
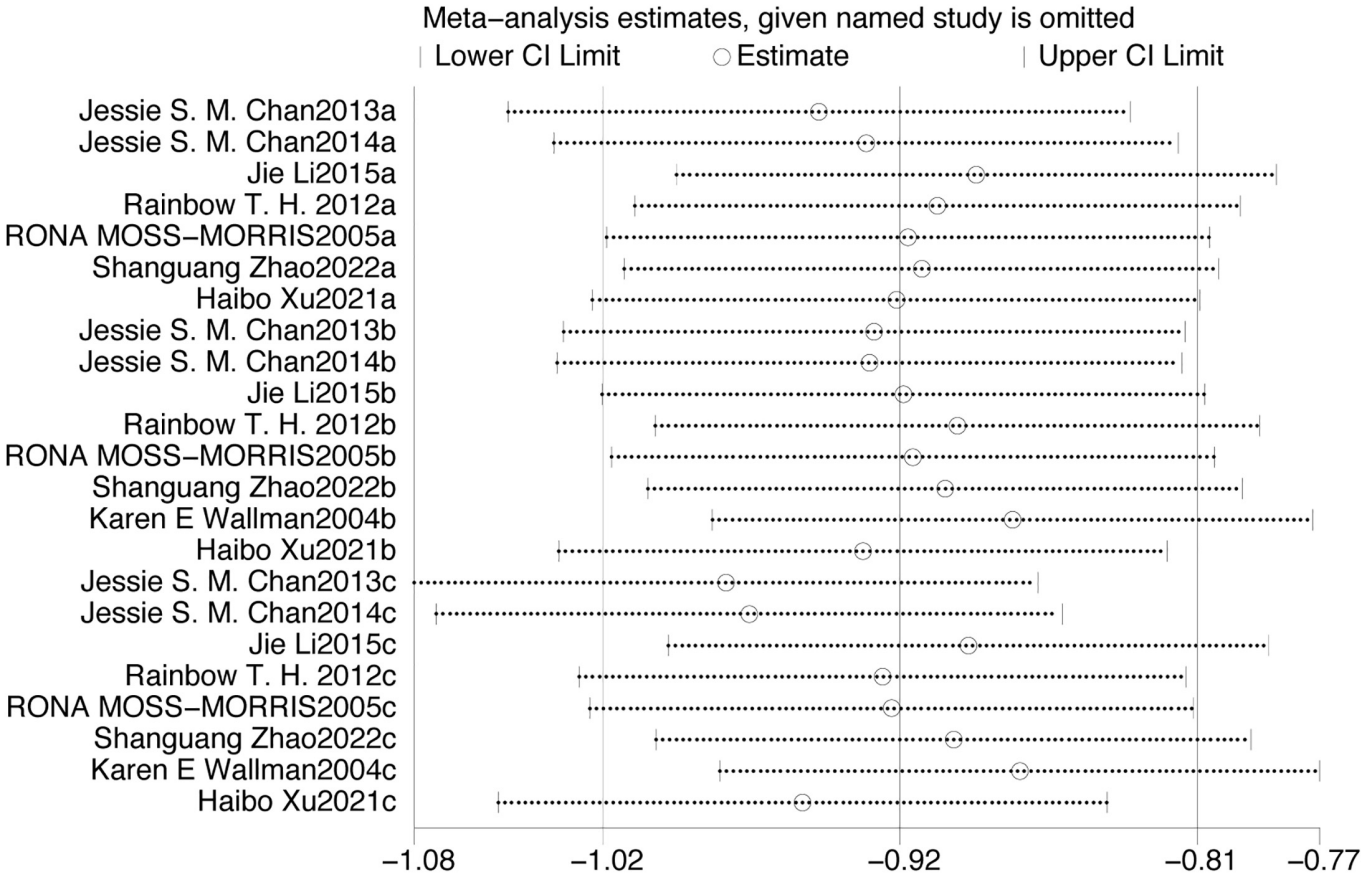
Sensitivity analysis of studies on exercise therapy for chronic fatigue syndrome.

In this study, 10 included studies used FS-14 as the primary outcome measure. Therefore, a funnel plot was generated to assess publication bias (Figure 10). The results indicated that the points did not exhibit left-right symmetry, indicating the presence of publication bias. On this basis, Egger's test was further conducted to assess publication bias. It yielded a *p*-value of < 0.0001, indicating significant publication bias. This may be related to no detailed categorization of intervention measures, inconsistent diagnostic criteria, and relatively small sample sizes in the included studies. As illustrated in Figure 11, all articles were within the specified range. Therefore, the results of the meta-analysis were robust.

## 4 Discussion

This study conducted a meta-analysis to evaluate the effectiveness and safety of exercise therapy for treating ME/CFS. A total of 13 studies are included, and three primary outcome measures were analyzed. The findings suggest that exercise therapy may provide overall clinical benefits for patients with ME/CFS. Additionally, exercise therapy significantly improved scores across various dimensions of FS-14 (9). However, scores for general fatigue, mental fatigue, and reduced motivation on MFI-20 (10) showed only a non-significant trend toward improvement. These results indicate that different measurement tools and fatigue dimensions vary in their sensitivity to exercise interventions. Therefore, future studies should focus on the selection and standardization of core outcome measures.

In terms of biological mechanisms, Xiong et al. (25) have found that the microbiome exhibits distinct stratification during the progression of disease. Notably, microbial dysbiosis is most pronounced in the early stages of ME/CFS and remains stable throughout the long-term course of illness. This characteristic may aid in identifying disease states. In pathophysiology, ME/CFS is categorized as a neurological disorder. In a study conducted by Wirth et al. (26), it is hypothesized that reduced cerebral blood flow, disturbances in local blood flow regulation and neurovascular coupling, central adrenal hyperactivity, hypocapnia, and elevated intracranial pressure appear to play crucial roles in the pathophysiology of neurological symptoms associated with ME/CFS. In summary, the pathophysiological mechanisms underlying ME/CFS remain undetermined, necessitating further studies for exploration. Existing research suggests that the effectiveness of exercise therapy may be based on a dual mechanism. Firstly, it provides psychological support aimed at motivating patients to overcome fear of activity and negative perceptions. Secondly, it systematically enhances physical health and exercise tolerance, thereby alleviating fatigue (27). Numerous studies, including the conclusions drawn by Larun et al. (5) support the findings of this study, indicating that exercise therapy is effective in alleviating fatigue more remarkably than the control group. In practice, it is important to note that inappropriate physical exercise that does not align with the patient's condition may cause harm. Therefore, ME/CFS patients should avoid independently going to gyms or engaging in blind training with the aim of self-healing. Exercise programs must be tailored to the individual's condition under the guidance of experts to avoid exacerbating the condition and ensure positive outcomes.

Subgroup analysis in this study suggested that different forms of exercise may alleviate fatigue symptoms in ME/CFS patients through distinct mechanisms. Conventional aerobic exercise appeared more effective in alleviating overall and physical fatigue, potentially through enhancing cardiovascular adaptation and muscle metabolic function. In contrast, Qigong (such as Baduanjin and Nine Turns to Longevity Practice) showed greater benefits for mental fatigue. This may be attributed to their core characteristics as mind–body exercises. Qigong involves slow, simple movements, with emphasis on focused attention and breath regulation. This practice can reduce sympathetic nervous tension and relieve psychological stress. Previous studies have indicated that telomere shortening and decreased telomerase activity are associated with ME/CFS and other chronic fatigue states. Qigong practice may not only reduce fatigue and improve physical function but also enhance telomerase activity. This provides a molecular-level explanation for its potential effects on alleviating mental fatigue and emotional symptoms, such as anxiety and depression. Therefore, it is recommended that future clinical practice tailor exercise interventions based on patients' predominant symptom profiles (such as primarily physical fatigue, primarily cognitive fatigue, or mixed type) to improve treatment precision and adherence (15–18).

Given that exercise interventions are difficult to fully blind, this study may be at risk of overestimating treatment effects. Accordingly, the risk of measurement bias was carefully considered during quality assessment, and all 13 included studies were rated as high risk for “other bias.” Publication bias was also specifically evaluated. Funnel plots suggested potential publication bias, which was further confirmed by Egger's test (*p* < 0.0001), indicating significant publication bias. This may further lead to an overestimation of the overall effect of exercise interventions. As this limitation is widespread in similar studies, its precise impact could not be quantified through sensitivity analyses or other statistical methods. Considering these factors, the study results should be interpreted with caution, and future high-quality studies with rigorous methodology and standardized procedures are warranted to provide more reliable evidence on the true effectiveness of exercise therapy.

This study evaluated a range of exercise-based complementary therapies for ME/CFS to enhance non-pharmacological treatment evidence and assess their efficacy and safety. Despite the comprehensiveness of this review, several limitations should be acknowledged: (i) geographic bias, as most studies were conducted in China; (ii) the inability to blind participants due to the nature of the interventions, potentially introducing performance and detection bias; (iii) inconsistent diagnostic criteria across studies; (iv) small sample sizes in certain trials; and (v) insufficient reporting of adverse events, which limits the assessment of safety.

## 5 Conclusion

This study suggests that exercise therapy is effective in reducing total fatigue, physical fatigue, and mental fatigue, while also alleviating reduced activity and motivation in daily life among patients with ME/CFS. It therefore provides significant clinical improvement and demonstrates multidimensional benefits. These findings support the integration of individualized exercise therapy into comprehensive management strategies for ME/CFS. However, due to limitations in the original study designs, these encouraging findings should still be considered preliminary rather than conclusive. Future research is expected to develop more objective biomarkers and outcome measures, implement rigorously standardized exercise interventions, and conduct larger, more rigorously designed clinical trials across different patient subgroups to provide more robust evidence for the treatment of ME/CFS.

## Data Availability

The original contributions presented in the study are included in the article/Supplementary material, further inquiries can be directed to the corresponding author.
