# Supplementary material for: Effectiveness and safety of exercise therapy in patients with myalgic encephalomyelitis/chronic fatigue syndrome: a meta-analysis
Source: Front Neurol. 2025 Nov 21;16:1681990. doi: 10.3389/fneur.2025.1681990 (PMC12678084; doi:10.3389/fneur.2025.1681990)
Supplement: Supplementary file 1 [file Supplementary_file_1.docx]

**Attachment 1** PubMed Search History

| Search number | Query | Sort By | Filters | Search Details | Results | Time |
| --- | --- | --- | --- | --- | --- | --- |
| 7 | (("Fatigue Syndrome, Chronic"[Mesh]) OR (((((((((((((((((((((Chronic Fatigue Syndromes[Title/Abstract]) OR (Chronic fatigue syndrome[Title/Abstract])) OR (Royal Free Disease[Title/Abstract])) OR (Chronic Fatigue[Title/Abstract] AND Immune Dysfunction Syndrome[Title/Abstract])) OR (Postviral Fatigue Syndrome[Title/Abstract])) OR (Postviral Fatigue Syndromes[Title/Abstract])) OR (Systemic Exertion Intolerance Disease[Title/Abstract])) OR (Myalgic Encephalomyelitis[Title/Abstract])) OR (Chronic Fatigue Syndrome[Title/Abstract])) OR (Chronic Fatigue Fibromyalgia Syndrome[Title/Abstract])) OR (Chronic Fatigue-Fibromyalgia Syndromes[Title/Abstract])) OR (Chronic Fatigue Disorder[Title/Abstract])) OR (Chronic Fatigue Disorders[Title/Abstract])) OR (Akureyri disease[Title/Abstract])) OR (benign myalgic encephalomyelitis[Title/Abstract])) OR (chronic fatigue[Title/Abstract])) OR (epidemic neuromyasthenia[Title/Abstract])) OR (fatigue syndrome[Title/Abstract])) OR (Iceland disease[Title/Abstract])) OR (syndrome of chronic fatigue[Title/Abstract])) OR (Yuppie flu[Title/Abstract]))) AND (("Exercise Therapy"[Mesh]) OR ((((((((((((((((((((corrective exercise[Title/Abstract]) OR (exercise movement techniques[Title/Abstract])) OR (exercise treatment[Title/Abstract])) OR (kinesiotherapeutic intervention[Title/Abstract])) OR (kinesiotherapeutic method[Title/Abstract])) OR (kinesiotherapeutic procedure[Title/Abstract])) OR (kinesiotherapeutic technique[Title/Abstract])) OR (kinesiotherapeutical treatment[Title/Abstract])) OR (kinesitherapeutic exercises[Title/Abstract])) OR (kinesitherapeutic intervention[Title/Abstract])) OR (kinesitherapeutic method[Title/Abstract])) OR (kinesitherapeutic methodology[Title/Abstract])) OR (kinesitherapeutic procedure[Title/Abstract])) OR (kinesitherapeutic technique[Title/Abstract])) OR (kinesitherapeutic treatment[Title/Abstract])) OR (kinesitherapeutical treatment[Title/Abstract])) OR (kinesitherapy[Title/Abstract])) OR (SKTM (specialized kinesitherapeutic methodology[Title/Abstract]))) OR (specialised kinesitherapeutic methodology[Title/Abstract])) OR (therapeutic exercise[Title/Abstract]))) |  |  | ("fatigue syndrome, chronic"[MeSH Terms] OR ("chronic fatigue syndromes"[Title/Abstract] OR "chronic fatigue syndrome"[Title/Abstract] OR "royal free disease"[Title/Abstract] OR ("chronic fatigue"[Title/Abstract] AND "immune dysfunction syndrome"[Title/Abstract]) OR "postviral fatigue syndrome"[Title/Abstract] OR "postviral fatigue syndromes"[Title/Abstract] OR "systemic exertion intolerance disease"[Title/Abstract] OR "myalgic encephalomyelitis"[Title/Abstract] OR "chronic fatigue syndrome"[Title/Abstract] OR ((("Chronic"[All Fields] OR "chronical"[All Fields] OR "chronically"[All Fields] OR "chronicities"[All Fields] OR "chronicity"[All Fields] OR "chronicization"[All Fields] OR "chronics"[All Fields]) AND ("fatiguability"[All Fields] OR "fatiguable"[All Fields] OR "Fatigue"[MeSH Terms] OR "Fatigue"[All Fields] OR "fatigued"[All Fields] OR "fatigues"[All Fields] OR "fatiguing"[All Fields] OR "fatigueability"[All Fields])) AND "fibromyalgia syndrome"[Title/Abstract]) OR "chronic fatigue fibromyalgia syndromes"[Title/Abstract] OR "chronic fatigue disorder"[Title/Abstract] OR "chronic fatigue disorders"[Title/Abstract] OR "akureyri disease"[Title/Abstract] OR "benign myalgic encephalomyelitis"[Title/Abstract] OR "chronic fatigue"[Title/Abstract] OR "epidemic neuromyasthenia"[Title/Abstract] OR "fatigue syndrome"[Title/Abstract] OR "iceland disease"[Title/Abstract] OR (("syndrom"[All Fields] OR "syndromal"[All Fields] OR "syndromally"[All Fields] OR "Syndrome"[MeSH Terms] OR "Syndrome"[All Fields] OR "Syndromes"[All Fields] OR "syndrome s"[All Fields] OR "syndromic"[All Fields] OR "syndroms"[All Fields]) AND "chronic fatigue"[Title/Abstract]) OR "yuppie flu"[Title/Abstract])) AND ("Exercise Therapy"[MeSH Terms] OR ("corrective exercise"[Title/Abstract] OR "exercise movement techniques"[Title/Abstract] OR "exercise treatment"[Title/Abstract] OR ("kinesiotherapeutic"[All Fields] AND "intervention"[Title/Abstract]) OR ("kinesiotherapeutic"[All Fields] AND "method"[Title/Abstract]) OR ("kinesiotherapeutic"[All Fields] AND "procedure"[Title/Abstract]) OR ("kinesiotherapeutic"[All Fields] AND "technique"[Title/Abstract]) OR ("kinesiotherapeutical"[All Fields] AND "treatment"[Title/Abstract]) OR "kinesitherapeutic exercises"[Title/Abstract] OR ("kinesitherapeutic"[All Fields] AND "intervention"[Title/Abstract]) OR ("kinesitherapeutic"[All Fields] AND "method"[Title/Abstract]) OR "kinesitherapeutic methodology"[Title/Abstract] OR ("kinesitherapeutic"[All Fields] AND "procedure"[Title/Abstract]) OR ("kinesitherapeutic"[All Fields] AND "technique"[Title/Abstract]) OR "kinesitherapeutic treatment"[Title/Abstract] OR ("treatment"[Title/Abstract]) OR "kinesitherapy"[Title/Abstract] OR ("SKTM"[All Fields] AND "specialized kinesitherapeutic methodology"[Title/Abstract]) OR (("medicine"[MeSH Terms] OR "medicine"[All Fields] OR "specialty"[All Fields] OR "special"[All Fields] OR "specialisations"[All Fields] OR "specialise"[All Fields] OR "specialised"[All Fields] OR "specialises"[All Fields] OR "specialising"[All Fields] OR "specialisms"[All Fields] OR "specialities"[All Fields] OR "speciality"[All Fields] OR "specialization"[MeSH Terms] OR "specialization"[All Fields] OR "specialisation"[All Fields] OR "specialism"[All Fields] OR "specializations"[All Fields] OR "specialize"[All Fields] OR "specialized"[All Fields] OR "specializes"[All Fields] OR "specializing"[All Fields] OR "specially"[All Fields] OR "specials"[All Fields] OR "specialties"[All Fields] OR "specialty s"[All Fields]) AND "kinesitherapeutic methodology"[Title/Abstract]) OR "therapeutic exercise"[Title/Abstract])) | 2,659 | 9:35:13 |
| 6 | ("Exercise Therapy"[Mesh]) OR ((((((((((((((((((((corrective exercise[Title/Abstract]) OR (exercise movement techniques[Title/Abstract])) OR (exercise treatment[Title/Abstract])) OR (kinesiotherapeutic intervention[Title/Abstract])) OR (kinesiotherapeutic method[Title/Abstract])) OR (kinesiotherapeutic procedure[Title/Abstract])) OR (kinesiotherapeutic technique[Title/Abstract])) OR (kinesiotherapeutical treatment[Title/Abstract])) OR (kinesitherapeutic exercises[Title/Abstract])) OR (kinesitherapeutic intervention[Title/Abstract])) OR (kinesitherapeutic method[Title/Abstract])) OR (kinesitherapeutic methodology[Title/Abstract])) OR (kinesitherapeutic procedure[Title/Abstract])) OR (kinesitherapeutic technique[Title/Abstract])) OR (kinesitherapeutic treatment[Title/Abstract])) OR (kinesitherapeutical treatment[Title/Abstract])) OR (kinesitherapy[Title/Abstract])) OR (SKTM (specialized kinesitherapeutic methodology[Title/Abstract]))) OR (specialised kinesitherapeutic methodology[Title/Abstract])) OR (therapeutic exercise[Title/Abstract])) |  |  | "Exercise Therapy"[MeSH Terms] OR ("corrective exercise"[Title/Abstract] OR "exercise movement techniques"[Title/Abstract] OR "exercise treatment"[Title/Abstract] OR ("kinesiotherapeutic"[All Fields] AND "intervention"[Title/Abstract]) OR ("kinesiotherapeutic"[All Fields] AND "method"[Title/Abstract]) OR ("kinesiotherapeutic"[All Fields] AND "procedure"[Title/Abstract]) OR ("kinesiotherapeutic"[All Fields] AND "technique"[Title/Abstract]) OR ("kinesiotherapeutical"[All Fields] AND "treatment"[Title/Abstract]) OR "kinesitherapeutic exercises"[Title/Abstract] OR ("kinesitherapeutic"[All Fields] AND "intervention"[Title/Abstract]) OR ("kinesitherapeutic"[All Fields] AND "method"[Title/Abstract]) OR "kinesitherapeutic methodology"[Title/Abstract] OR ("kinesitherapeutic"[All Fields] AND "procedure"[Title/Abstract]) OR ("kinesitherapeutic"[All Fields] AND "technique"[Title/Abstract]) OR "kinesitherapeutic treatment"[Title/Abstract] OR ("treatment"[Title/Abstract]) OR "kinesitherapy"[Title/Abstract] OR ("SKTM"[All Fields] AND "specialized kinesitherapeutic methodology"[Title/Abstract]) OR (("medicine"[MeSH Terms] OR "medicine"[All Fields] OR "specialty"[All Fields] OR "special"[All Fields] OR "specialisations"[All Fields] OR "specialise"[All Fields] OR "specialised"[All Fields] OR "specialises"[All Fields] OR "specialising"[All Fields] OR "specialisms"[All Fields] OR "specialities"[All Fields] OR "speciality"[All Fields] OR "specialization"[MeSH Terms] OR "specialization"[All Fields] OR "specialisation"[All Fields] OR "specialism"[All Fields] OR "specializations"[All Fields] OR "specialize"[All Fields] OR "specialized"[All Fields] OR "specializes"[All Fields] OR "specializing"[All Fields] OR "specially"[All Fields] OR "specials"[All Fields] OR "specialties"[All Fields] OR "specialty s"[All Fields]) AND "kinesitherapeutic methodology"[Title/Abstract]) OR "therapeutic exercise"[Title/Abstract]) | 5,467,886 | 9:34:42 |
| 5 | (((((((((((((((((((corrective exercise[Title/Abstract]) OR (exercise movement techniques[Title/Abstract])) OR (exercise treatment[Title/Abstract])) OR (kinesiotherapeutic intervention[Title/Abstract])) OR (kinesiotherapeutic method[Title/Abstract])) OR (kinesiotherapeutic procedure[Title/Abstract])) OR (kinesiotherapeutic technique[Title/Abstract])) OR (kinesiotherapeutical treatment[Title/Abstract])) OR (kinesitherapeutic exercises[Title/Abstract])) OR (kinesitherapeutic intervention[Title/Abstract])) OR (kinesitherapeutic method[Title/Abstract])) OR (kinesitherapeutic methodology[Title/Abstract])) OR (kinesitherapeutic procedure[Title/Abstract])) OR (kinesitherapeutic technique[Title/Abstract])) OR (kinesitherapeutic treatment[Title/Abstract])) OR (kinesitherapeutical treatment[Title/Abstract])) OR (kinesitherapy[Title/Abstract])) OR (SKTM (specialized kinesitherapeutic methodology[Title/Abstract]))) OR (specialised kinesitherapeutic methodology[Title/Abstract])) OR (therapeutic exercise[Title/Abstract]) |  |  | "corrective exercise"[Title/Abstract] OR "exercise movement techniques"[Title/Abstract] OR "exercise treatment"[Title/Abstract] OR ("kinesiotherapeutic"[All Fields] AND "intervention"[Title/Abstract]) OR ("kinesiotherapeutic"[All Fields] AND "method"[Title/Abstract]) OR ("kinesiotherapeutic"[All Fields] AND "procedure"[Title/Abstract]) OR ("kinesiotherapeutic"[All Fields] AND "technique"[Title/Abstract]) OR ("kinesiotherapeutical"[All Fields] AND "treatment"[Title/Abstract]) OR "kinesitherapeutic exercises"[Title/Abstract] OR ("kinesitherapeutic"[All Fields] AND "intervention"[Title/Abstract]) OR ("kinesitherapeutic"[All Fields] AND "method"[Title/Abstract]) OR "kinesitherapeutic methodology"[Title/Abstract] OR ("kinesitherapeutic"[All Fields] AND "procedure"[Title/Abstract]) OR ("kinesitherapeutic"[All Fields] AND "technique"[Title/Abstract]) OR "kinesitherapeutic treatment"[Title/Abstract] OR ("treatment"[Title/Abstract]) OR "kinesitherapy"[Title/Abstract] OR ("SKTM"[All Fields] AND "specialized kinesitherapeutic methodology"[Title/Abstract]) OR (("medicine"[MeSH Terms] OR "medicine"[All Fields] OR "specialty"[All Fields] OR "special"[All Fields] OR "specialisations"[All Fields] OR "specialise"[All Fields] OR "specialised"[All Fields] OR "specialises"[All Fields] OR "specialising"[All Fields] OR "specialisms"[All Fields] OR "specialities"[All Fields] OR "speciality"[All Fields] OR "specialization"[MeSH Terms] OR "specialization"[All Fields] OR "specialisation"[All Fields] OR "specialism"[All Fields] OR "specializations"[All Fields] OR "specialize"[All Fields] OR "specialized"[All Fields] OR "specializes"[All Fields] OR "specializing"[All Fields] OR "specially"[All Fields] OR "specials"[All Fields] OR "specialties"[All Fields] OR "specialty s"[All Fields]) AND "kinesitherapeutic methodology"[Title/Abstract]) OR "therapeutic exercise"[Title/Abstract] | 5,420,157 | 9:34:14 |
| 4 | "Exercise Therapy"[Mesh] | Most Recent |  | "Exercise Therapy"[MeSH Terms] | 65,159 | 9:26:49 |
| 3 | ("Fatigue Syndrome, Chronic"[Mesh]) OR (((((((((((((((((((((Chronic Fatigue Syndromes[Title/Abstract]) OR (Chronic fatigue syndrome[Title/Abstract])) OR (Royal Free Disease[Title/Abstract])) OR (Chronic Fatigue[Title/Abstract] AND Immune Dysfunction Syndrome[Title/Abstract])) OR (Postviral Fatigue Syndrome[Title/Abstract])) OR (Postviral Fatigue Syndromes[Title/Abstract])) OR (Systemic Exertion Intolerance Disease[Title/Abstract])) OR (Myalgic Encephalomyelitis[Title/Abstract])) OR (Chronic Fatigue Syndrome[Title/Abstract])) OR (Chronic Fatigue Fibromyalgia Syndrome[Title/Abstract])) OR (Chronic Fatigue-Fibromyalgia Syndromes[Title/Abstract])) OR (Chronic Fatigue Disorder[Title/Abstract])) OR (Chronic Fatigue Disorders[Title/Abstract])) OR (Akureyri disease[Title/Abstract])) OR (benign myalgic encephalomyelitis[Title/Abstract])) OR (chronic fatigue[Title/Abstract])) OR (epidemic neuromyasthenia[Title/Abstract])) OR (fatigue syndrome[Title/Abstract])) OR (Iceland disease[Title/Abstract])) OR (syndrome of chronic fatigue[Title/Abstract])) OR (Yuppie flu[Title/Abstract])) |  |  | "fatigue syndrome, chronic"[MeSH Terms] OR ("chronic fatigue syndromes"[Title/Abstract] OR "chronic fatigue syndrome"[Title/Abstract] OR "royal free disease"[Title/Abstract] OR ("chronic fatigue"[Title/Abstract] AND "immune dysfunction syndrome"[Title/Abstract]) OR "postviral fatigue syndrome"[Title/Abstract] OR "postviral fatigue syndromes"[Title/Abstract] OR "systemic exertion intolerance disease"[Title/Abstract] OR "myalgic encephalomyelitis"[Title/Abstract] OR "chronic fatigue syndrome"[Title/Abstract] OR ((("Chronic"[All Fields] OR "chronical"[All Fields] OR "chronically"[All Fields] OR "chronicities"[All Fields] OR "chronicity"[All Fields] OR "chronicization"[All Fields] OR "chronics"[All Fields]) AND ("fatiguability"[All Fields] OR "fatiguable"[All Fields] OR "Fatigue"[MeSH Terms] OR "Fatigue"[All Fields] OR "fatigued"[All Fields] OR "fatigues"[All Fields] OR "fatiguing"[All Fields] OR "fatigueability"[All Fields])) AND "fibromyalgia syndrome"[Title/Abstract]) OR "chronic fatigue fibromyalgia syndromes"[Title/Abstract] OR "chronic fatigue disorder"[Title/Abstract] OR "chronic fatigue disorders"[Title/Abstract] OR "akureyri disease"[Title/Abstract] OR "benign myalgic encephalomyelitis"[Title/Abstract] OR "chronic fatigue"[Title/Abstract] OR "epidemic neuromyasthenia"[Title/Abstract] OR "fatigue syndrome"[Title/Abstract] OR "iceland disease"[Title/Abstract] OR (("syndrom"[All Fields] OR "syndromal"[All Fields] OR "syndromally"[All Fields] OR "Syndrome"[MeSH Terms] OR "Syndrome"[All Fields] OR "Syndromes"[All Fields] OR "syndrome s"[All Fields] OR "syndromic"[All Fields] OR "syndroms"[All Fields]) AND "chronic fatigue"[Title/Abstract]) OR "yuppie flu"[Title/Abstract]) | 10,362 | 9:25:52 |
| 2 | ((((((((((((((((((((Chronic Fatigue Syndromes[Title/Abstract]) OR (Chronic fatigue syndrome[Title/Abstract])) OR (Royal Free Disease[Title/Abstract])) OR (Chronic Fatigue[Title/Abstract] AND Immune Dysfunction Syndrome[Title/Abstract])) OR (Postviral Fatigue Syndrome[Title/Abstract])) OR (Postviral Fatigue Syndromes[Title/Abstract])) OR (Systemic Exertion Intolerance Disease[Title/Abstract])) OR (Myalgic Encephalomyelitis[Title/Abstract])) OR (Chronic Fatigue Syndrome[Title/Abstract])) OR (Chronic Fatigue Fibromyalgia Syndrome[Title/Abstract])) OR (Chronic Fatigue-Fibromyalgia Syndromes[Title/Abstract])) OR (Chronic Fatigue Disorder[Title/Abstract])) OR (Chronic Fatigue Disorders[Title/Abstract])) OR (Akureyri disease[Title/Abstract])) OR (benign myalgic encephalomyelitis[Title/Abstract])) OR (chronic fatigue[Title/Abstract])) OR (epidemic neuromyasthenia[Title/Abstract])) OR (fatigue syndrome[Title/Abstract])) OR (Iceland disease[Title/Abstract])) OR (syndrome of chronic fatigue[Title/Abstract])) OR (Yuppie flu[Title/Abstract]) |  |  | "chronic fatigue syndromes"[Title/Abstract] OR "chronic fatigue syndrome"[Title/Abstract] OR "royal free disease"[Title/Abstract] OR ("chronic fatigue"[Title/Abstract] AND "immune dysfunction syndrome"[Title/Abstract]) OR "postviral fatigue syndrome"[Title/Abstract] OR "postviral fatigue syndromes"[Title/Abstract] OR "systemic exertion intolerance disease"[Title/Abstract] OR "myalgic encephalomyelitis"[Title/Abstract] OR "chronic fatigue syndrome"[Title/Abstract] OR ((("Chronic"[All Fields] OR "chronical"[All Fields] OR "chronically"[All Fields] OR "chronicities"[All Fields] OR "chronicity"[All Fields] OR "chronicization"[All Fields] OR "chronics"[All Fields]) AND ("fatiguability"[All Fields] OR "fatiguable"[All Fields] OR "Fatigue"[MeSH Terms] OR "Fatigue"[All Fields] OR "fatigued"[All Fields] OR "fatigues"[All Fields] OR "fatiguing"[All Fields] OR "fatigueability"[All Fields])) AND "fibromyalgia syndrome"[Title/Abstract]) OR "chronic fatigue fibromyalgia syndromes"[Title/Abstract] OR "chronic fatigue disorder"[Title/Abstract] OR "chronic fatigue disorders"[Title/Abstract] OR "akureyri disease"[Title/Abstract] OR "benign myalgic encephalomyelitis"[Title/Abstract] OR "chronic fatigue"[Title/Abstract] OR "epidemic neuromyasthenia"[Title/Abstract] OR "fatigue syndrome"[Title/Abstract] OR "iceland disease"[Title/Abstract] OR (("syndrom"[All Fields] OR "syndromal"[All Fields] OR "syndromally"[All Fields] OR "Syndrome"[MeSH Terms] OR "Syndrome"[All Fields] OR "Syndromes"[All Fields] OR "syndrome s"[All Fields] OR "syndromic"[All Fields] OR "syndroms"[All Fields]) AND "chronic fatigue"[Title/Abstract]) OR "yuppie flu"[Title/Abstract] | 9,524 | 9:24:47 |
| 1 | "Fatigue Syndrome, Chronic"[Mesh] | Most Recent |  | "fatigue syndrome, chronic"[MeSH Terms] | 6,304 | 9:20:43 |

**Attachment 2** Web of science search strategy

| Entitlements | # | Search Query | Database | Results | Date Run |
| --- | --- | --- | --- | --- | --- |
| - WOS.IC: 1993 to 2024 - WOS.CCR: 1985 to 2024 - WOS.SCI: 1975 to 2024 - WOS.AHCI: 1975 to 2024 - WOS.BHCI: 2005 to 2024 - WOS.BSCI: 2005 to 2024 - WOS.ESCI: 2019 to 2024 - WOS.ISTP: 1990 to 2024 - WOS.SSCI: 1965 to 2024 - WOS.ISSHP: 1990 to 2024 | 1 | Chronic fatigue syndrome (Topic) OR Chronic Fatigue Syndromes (Topic) OR Royal Free Disease (Topic) OR Chronic Fatigue and Immune Dysfunction Syndrome (Topic) OR Postviral Fatigue Syndrome (Topic) OR Postviral Fatigue Syndromes (Topic) OR Systemic Exertion Intolerance Disease (Topic) OR Myalgic Encephalomyelitis (Topic) OR Chronic Fatigue Syndrome (Topic) OR Chronic Fatigue Fibromyalgia Syndrome (Topic) OR Chronic Fatigue-Fibromyalgia Syndromes (Topic) OR Chronic Fatigue Disorder (Topic) OR Chronic Fatigue Disorders (Topic) OR Akureyri disease (Topic) OR benign myalgic encephalomyelitis (Topic) OR chronic fatigue (Topic) OR epidemic neuromyasthenia (Topic) OR fatigue syndrome (Topic) OR Iceland disease (Topic) OR syndrome of chronic fatigue (Topic) OR Yuppie flu (Topic) | Web of Science Core Collection | 37437 | Mon Mar 04 2024 21:16:49 GMT+0800 (China Standard Time) |
| - WOS.IC: 1993 to 2024 - WOS.CCR: 1985 to 2024 - WOS.SCI: 1975 to 2024 - WOS.AHCI: 1975 to 2024 - WOS.BHCI: 2005 to 2024 - WOS.BSCI: 2005 to 2024 - WOS.ESCI: 2019 to 2024 - WOS.ISTP: 1990 to 2024 - WOS.SSCI: 1965 to 2024 - WOS.ISSHP: 1990 to 2024 | 2 | (((((((((((((((((((((TS=(Exercise therapy)) OR TS=(kinesiotherapy)) OR TS=(corrective exercise)) OR TS=(exercise movement techniques)) OR TS=(exercise treatment)) OR TS=(kinesiotherapeutic intervention)) OR TS=(kinesiotherapeutic method)) OR TS=(kinesiotherapeutic procedure)) OR TS=(kinesiotherapeutic technique)) OR TS=(kinesiotherapeutical treatment)) OR TS=(kinesitherapeutic exercises)) OR TS=(kinesitherapeutic intervention)) OR TS=(kinesitherapeutic method)) OR TS=(kinesitherapeutic methodology)) OR TS=(kinesitherapeutic procedure)) OR TS=(kinesitherapeutic technique)) OR TS=(kinesitherapeutic treatment)) OR TS=(kinesitherapeutical treatment)) OR TS=(kinesitherapy)) OR TS=(SKTM (specialized kinesitherapeutic methodology))) OR TS=(specialised kinesitherapeutic methodology)) OR TS=(therapeutic exercise) | Web of Science Core Collection | 123467 | Mon Mar 04 2024 21:20:41 GMT+0800 (China Standard Time) |
| - WOS.IC: 1993 to 2024 - WOS.CCR: 1985 to 2024 - WOS.SCI: 1975 to 2024 - WOS.AHCI: 1975 to 2024 - WOS.BHCI: 2005 to 2024 - WOS.BSCI: 2005 to 2024 - WOS.ESCI: 2019 to 2024 - WOS.ISTP: 1990 to 2024 - WOS.SSCI: 1965 to 2024 - WOS.ISSHP: 1990 to 2024 | 3 | #2 AND #1 | Web of Science Core Collection | 2376 | Mon Mar 04 2024 21:21:24 GMT+0800 (China Standard Time) |

**Attachment 3** Embase

Session Results

.......................................................

No. Query Results Results Date

#7. #3 AND #6 787 4 Mar 2024

#6. #4 OR #5 108,450 4 Mar 2024

#5. 'corrective exercise':ab,ti OR 'exercise movement 4,124 4 Mar 2024

techniques':ab,ti OR 'exercise treatment':ab,ti

OR 'kinesiotherapeutic intervention':ab,ti OR

'kinesiotherapeutic method':ab,ti OR

'kinesiotherapeutic technique':ab,ti OR

'kinesiotherapeutic procedure':ab,ti OR

'kinesiotherapeutical treatment':ab,ti OR

'kinesitherapeutic exercises':ab,ti OR

'kinesitherapeutic intervention':ab,ti OR

'kinesitherapeutic method':ab,ti OR

'kinesitherapeutic methodology':ab,ti OR

'kinesitherapeutic procedure':ab,ti OR

'kinesitherapeutic technique':ab,ti OR

'kinesitherapeutic treatment':ab,ti OR

'kinesitherapeutical treatment':ab,ti OR

kinesitherapy:ab,ti OR (sktm:ab,ti AND

'specialized kinesitherapeutic

methodology':ab,ti) OR 'specialised

kinesitherapeutic methodology':ab,ti OR

'specialized kinesitherapeutic methodology':ab,ti

OR 'therapeutic exercise':ab,ti

#4. 'kinesiotherapy'/exp OR 'kinesiotherapy' 107,003 4 Mar 2024

#3. #1 OR #2 22,367 4 Mar 2024

#2. 'chronic fatigue syndromes':ab,ti OR 'royal free 12,057 4 Mar 2024

disease':ab,ti OR ('chronic fatigue':ab,ti AND

'immune dysfunction syndrome':ab,ti) OR

'postviral fatigue syndrome':ab,ti OR 'postviral

fatigue syndromes':ab,ti OR 'systemic exertion

intolerance disease':ab,ti OR 'myalgic

encephalomyelitis':ab,ti OR 'chronic fatigue

syndrome':ab,ti OR 'chronic fatigue fibromyalgia

syndrome':ab,ti OR 'chronic fatigue-fibromyalgia

syndromes':ab,ti OR 'chronic fatigue

disorder':ab,ti OR 'chronic fatigue

disorders':ab,ti OR 'akureyri disease':ab,ti OR

'benign myalgic encephalomyelitis':ab,ti OR

'chronic fatigue':ab,ti OR 'epidemic

neuromyasthenia':ab,ti OR 'fatigue

syndrome':ab,ti OR 'iceland disease':ab,ti OR

'syndrome of chronic fatigue':ab,ti OR 'yuppie

flu':ab,ti

#1. 'chronic fatigue syndrome'/exp OR 'chronic 20,290 4 Mar 2024

fatigue syndrome'

.......................................................

**Attachment 4** Chocrane

Search Name:

Date Run: 29/02/2024 07:23:30

Comment:

| ID | Search | Hits |
| --- | --- | --- |
| #1 | MeSH descriptor: [Fatigue Syndrome, Chronic] explode all trees | 550 |
| #2 | (Chronic Fatigue Syndromes):ti,ab,kw OR (Royal Free Disease):ti,ab,kw OR (Chronic Fatigue and Immune Dysfunction Syndrome):ti,ab,kw OR (Postviral Fatigue Syndrome):ti,ab,kw OR (Postviral Fatigue Syndromes):ti,ab,kw (Word variations have been searched) | 2425 |
| #3 | (Systemic Exertion Intolerance Disease):ti,ab,kw OR (Myalgic Encephalomyelitis):ti,ab,kw OR (Chronic Fatigue Syndrome):ti,ab,kw OR (Chronic Fatigue Fibromyalgia Syndrome):ti,ab,kw OR (Chronic Fatigue-Fibromyalgia Syndromes):ti,ab,kw (Word variations have been searched) | 2342 |
| #4 | (Chronic Fatigue Disorder):ti,ab,kw OR (Chronic Fatigue Disorders):ti,ab,kw OR (Akureyri disease):ti,ab,kw OR (benign myalgic encephalomyelitis):ti,ab,kw OR (chronic fatigue):ti,ab,kw (Word variations have been searched) | 8361 |
| #5 | (epidemic neuromyasthenia):ti,ab,kw OR (fatigue syndrome):ti,ab,kw OR (Iceland disease):ti,ab,kw OR (syndrome of chronic fatigue):ti,ab,kw OR (Yuppie flu):ti,ab,kw (Word variations have been searched) | 6756 |
| #6 | #1 OR #2 OR #3 OR #4 OR #5 | 12944 |
| #7 | MeSH descriptor: [Exercise Therapy] explode all trees | 21541 |
| #8 | (kinesiotherapy):ti,ab,kw OR (corrective exercise):ti,ab,kw OR (exercise movement techniques):ti,ab,kw OR (exercise treatment):ti,ab,kw OR (kinesiotherapeutic intervention):ti,ab,kw (Word variations have been searched) | 60303 |
| #9 | (kinesiotherapeutic method):ti,ab,kw OR (kinesiotherapeutic procedure):ti,ab,kw OR (kinesiotherapeutic technique):ti,ab,kw OR (kinesiotherapeutical treatment):ti,ab,kw OR (kinesitherapeutic exercises):ti,ab,kw (Word variations have been searched) | 14 |
| #10 | (kinesitherapeutic intervention):ti,ab,kw OR (kinesitherapeutic methodology):ti,ab,kw OR (kinesitherapeutic method):ti,ab,kw OR (kinesitherapeutic procedure):ti,ab,kw OR (kinesitherapeutic technique):ti,ab,kw (Word variations have been searched) | 9 |
| #11 | (kinesitherapeutic treatment):ti,ab,kw OR (kinesitherapeutical treatment):ti,ab,kw OR (kinesitherapy):ti,ab,kw OR (SKTM (specialized kinesitherapeutic methodology)):ti,ab,kw OR (specialised kinesitherapeutic methodology):ti,ab,kw (Word variations have been searched) | 123 |
| #12 | (specialized kinesitherapeutic methodology):ti,ab,kw OR (therapeutic exercise):ti,ab,kw (Word variations have been searched) | 16136 |
| #13 | #7 OR #8 OR #9 OR #10 OR #11 OR #12 | 75013 |
| #14 | #6 AND #13 | 1724 |

**Attachment 5** PubMed Search History(2025.06.07)

| Search number | Query | Sort By | Filters | Search Details | Results | Time |
| --- | --- | --- | --- | --- | --- | --- |
| 5 | ((("Fatigue Syndrome, Chronic"[Mesh]) OR (((((((((((((((((((((Chronic Fatigue Syndromes[Title/Abstract]) OR (Chronic fatigue syndrome[Title/Abstract])) OR (Royal Free Disease[Title/Abstract])) OR (Chronic Fatigue[Title/Abstract] AND Immune Dysfunction Syndrome[Title/Abstract])) OR (Postviral Fatigue Syndrome[Title/Abstract])) OR (Postviral Fatigue Syndromes[Title/Abstract])) OR (Systemic Exertion Intolerance Disease[Title/Abstract])) OR (Myalgic Encephalomyelitis[Title/Abstract])) OR (Chronic Fatigue Syndrome[Title/Abstract])) OR (Chronic Fatigue Fibromyalgia Syndrome[Title/Abstract])) OR (Chronic Fatigue-Fibromyalgia Syndromes[Title/Abstract])) OR (Chronic Fatigue Disorder[Title/Abstract])) OR (Chronic Fatigue Disorders[Title/Abstract])) OR (Akureyri disease[Title/Abstract])) OR (benign myalgic encephalomyelitis[Title/Abstract])) OR (chronic fatigue[Title/Abstract])) OR (epidemic neuromyasthenia[Title/Abstract])) OR (fatigue syndrome[Title/Abstract])) OR (Iceland disease[Title/Abstract])) OR (syndrome of chronic fatigue[Title/Abstract])) OR (Yuppie flu[Title/Abstract]))) AND ((((((((((((((((((((corrective exercise[Title/Abstract]) OR (exercise movement techniques[Title/Abstract])) OR (exercise treatment[Title/Abstract])) OR (kinesiotherapeutic intervention[Title/Abstract])) OR (kinesiotherapeutic method[Title/Abstract])) OR (kinesiotherapeutic procedure[Title/Abstract])) OR (kinesiotherapeutic technique[Title/Abstract])) OR (kinesiotherapeutical treatment[Title/Abstract])) OR (kinesitherapeutic exercises[Title/Abstract])) OR (kinesitherapeutic intervention[Title/Abstract])) OR (kinesitherapeutic method[Title/Abstract])) OR (kinesitherapeutic methodology[Title/Abstract])) OR (kinesitherapeutic procedure[Title/Abstract])) OR (kinesitherapeutic technique[Title/Abstract])) OR (kinesitherapeutic treatment[Title/Abstract])) OR (kinesitherapeutical treatment[Title/Abstract])) OR (kinesitherapy[Title/Abstract])) OR (SKTM (specialized kinesitherapeutic methodology[Title/Abstract]))) OR (specialised kinesitherapeutic methodology[Title/Abstract])) OR (therapeutic exercise[Title/Abstract]))) AND (("2024/4/1"[Date - Publication] : "3000"[Date - Publication])) |  |  | ("fatigue syndrome, chronic"[MeSH Terms] OR ("chronic fatigue syndromes"[Title/Abstract] OR "chronic fatigue syndrome"[Title/Abstract] OR "royal free disease"[Title/Abstract] OR ("chronic fatigue"[Title/Abstract] AND "immune dysfunction syndrome"[Title/Abstract]) OR "postviral fatigue syndrome"[Title/Abstract] OR "postviral fatigue syndromes"[Title/Abstract] OR "systemic exertion intolerance disease"[Title/Abstract] OR "myalgic encephalomyelitis"[Title/Abstract] OR "chronic fatigue syndrome"[Title/Abstract] OR ((("Chronic"[All Fields] OR "chronical"[All Fields] OR "chronically"[All Fields] OR "chronicities"[All Fields] OR "chronicity"[All Fields] OR "chronicization"[All Fields] OR "chronics"[All Fields]) AND ("fatiguability"[All Fields] OR "fatiguable"[All Fields] OR "Fatigue"[MeSH Terms] OR "Fatigue"[All Fields] OR "fatigued"[All Fields] OR "fatigues"[All Fields] OR "fatiguing"[All Fields] OR "fatigueability"[All Fields])) AND "fibromyalgia syndrome"[Title/Abstract]) OR "chronic fatigue fibromyalgia syndromes"[Title/Abstract] OR "chronic fatigue disorder"[Title/Abstract] OR "chronic fatigue disorders"[Title/Abstract] OR "akureyri disease"[Title/Abstract] OR "benign myalgic encephalomyelitis"[Title/Abstract] OR "chronic fatigue"[Title/Abstract] OR "epidemic neuromyasthenia"[Title/Abstract] OR "fatigue syndrome"[Title/Abstract] OR "iceland disease"[Title/Abstract] OR (("syndrom"[All Fields] OR "syndromal"[All Fields] OR "syndromally"[All Fields] OR "Syndrome"[MeSH Terms] OR "Syndrome"[All Fields] OR "Syndromes"[All Fields] OR "syndrome s"[All Fields] OR "syndromic"[All Fields] OR "syndroms"[All Fields]) AND "chronic fatigue"[Title/Abstract]) OR "yuppie flu"[Title/Abstract])) AND ("corrective exercise"[Title/Abstract] OR "exercise movement techniques"[Title/Abstract] OR "exercise treatment"[Title/Abstract] OR ("kinesiotherapeutic"[All Fields] AND "intervention"[Title/Abstract]) OR ("kinesiotherapeutic"[All Fields] AND "method"[Title/Abstract]) OR ("kinesiotherapeutic"[All Fields] AND "procedure"[Title/Abstract]) OR ("kinesiotherapeutic"[All Fields] AND "technique"[Title/Abstract]) OR ("kinesiotherapeutical"[All Fields] AND "treatment"[Title/Abstract]) OR "kinesitherapeutic exercises"[Title/Abstract] OR ("kinesitherapeutic"[All Fields] AND "intervention"[Title/Abstract]) OR ("kinesitherapeutic"[All Fields] AND "method"[Title/Abstract]) OR "kinesitherapeutic methodology"[Title/Abstract] OR ("kinesitherapeutic"[All Fields] AND "procedure"[Title/Abstract]) OR ("kinesitherapeutic"[All Fields] AND "technique"[Title/Abstract]) OR "kinesitherapeutic treatment"[Title/Abstract] OR ("treatment"[Title/Abstract]) OR "kinesitherapy"[Title/Abstract] OR ("SKTM"[All Fields] AND "specialized kinesitherapeutic methodology"[Title/Abstract]) OR (("medicine"[MeSH Terms] OR "medicine"[All Fields] OR "specialty"[All Fields] OR "special"[All Fields] OR "specialisations"[All Fields] OR "specialise"[All Fields] OR "specialised"[All Fields] OR "specialises"[All Fields] OR "specialising"[All Fields] OR "specialisms"[All Fields] OR "specialities"[All Fields] OR "speciality"[All Fields] OR "specialization"[MeSH Terms] OR "specialization"[All Fields] OR "specialisation"[All Fields] OR "specialism"[All Fields] OR "specializations"[All Fields] OR "specialize"[All Fields] OR "specialized"[All Fields] OR "specializes"[All Fields] OR "specializing"[All Fields] OR "specially"[All Fields] OR "specials"[All Fields] OR "specialties"[All Fields] OR "specialty s"[All Fields]) AND "kinesitherapeutic methodology"[Title/Abstract]) OR "therapeutic exercise"[Title/Abstract]) AND 2024/04/01:3000/12/31[Date - Publication] | 219 | 9:29:51 |
| 4 | ("2024/4/1"[Date - Publication] : "3000"[Date - Publication]) |  |  | 2024/04/01:3000/12/31[Date - Publication] | 2,012,678 | 9:28:54 |
| 3 | (("Fatigue Syndrome, Chronic"[Mesh]) OR (((((((((((((((((((((Chronic Fatigue Syndromes[Title/Abstract]) OR (Chronic fatigue syndrome[Title/Abstract])) OR (Royal Free Disease[Title/Abstract])) OR (Chronic Fatigue[Title/Abstract] AND Immune Dysfunction Syndrome[Title/Abstract])) OR (Postviral Fatigue Syndrome[Title/Abstract])) OR (Postviral Fatigue Syndromes[Title/Abstract])) OR (Systemic Exertion Intolerance Disease[Title/Abstract])) OR (Myalgic Encephalomyelitis[Title/Abstract])) OR (Chronic Fatigue Syndrome[Title/Abstract])) OR (Chronic Fatigue Fibromyalgia Syndrome[Title/Abstract])) OR (Chronic Fatigue-Fibromyalgia Syndromes[Title/Abstract])) OR (Chronic Fatigue Disorder[Title/Abstract])) OR (Chronic Fatigue Disorders[Title/Abstract])) OR (Akureyri disease[Title/Abstract])) OR (benign myalgic encephalomyelitis[Title/Abstract])) OR (chronic fatigue[Title/Abstract])) OR (epidemic neuromyasthenia[Title/Abstract])) OR (fatigue syndrome[Title/Abstract])) OR (Iceland disease[Title/Abstract])) OR (syndrome of chronic fatigue[Title/Abstract])) OR (Yuppie flu[Title/Abstract]))) AND ((((((((((((((((((((corrective exercise[Title/Abstract]) OR (exercise movement techniques[Title/Abstract])) OR (exercise treatment[Title/Abstract])) OR (kinesiotherapeutic intervention[Title/Abstract])) OR (kinesiotherapeutic method[Title/Abstract])) OR (kinesiotherapeutic procedure[Title/Abstract])) OR (kinesiotherapeutic technique[Title/Abstract])) OR (kinesiotherapeutical treatment[Title/Abstract])) OR (kinesitherapeutic exercises[Title/Abstract])) OR (kinesitherapeutic intervention[Title/Abstract])) OR (kinesitherapeutic method[Title/Abstract])) OR (kinesitherapeutic methodology[Title/Abstract])) OR (kinesitherapeutic procedure[Title/Abstract])) OR (kinesitherapeutic technique[Title/Abstract])) OR (kinesitherapeutic treatment[Title/Abstract])) OR (kinesitherapeutical treatment[Title/Abstract])) OR (kinesitherapy[Title/Abstract])) OR (SKTM (specialized kinesitherapeutic methodology[Title/Abstract]))) OR (specialised kinesitherapeutic methodology[Title/Abstract])) OR (therapeutic exercise[Title/Abstract])) |  |  | ("fatigue syndrome, chronic"[MeSH Terms] OR ("chronic fatigue syndromes"[Title/Abstract] OR "chronic fatigue syndrome"[Title/Abstract] OR "royal free disease"[Title/Abstract] OR ("chronic fatigue"[Title/Abstract] AND "immune dysfunction syndrome"[Title/Abstract]) OR "postviral fatigue syndrome"[Title/Abstract] OR "postviral fatigue syndromes"[Title/Abstract] OR "systemic exertion intolerance disease"[Title/Abstract] OR "myalgic encephalomyelitis"[Title/Abstract] OR "chronic fatigue syndrome"[Title/Abstract] OR ((("Chronic"[All Fields] OR "chronical"[All Fields] OR "chronically"[All Fields] OR "chronicities"[All Fields] OR "chronicity"[All Fields] OR "chronicization"[All Fields] OR "chronics"[All Fields]) AND ("fatiguability"[All Fields] OR "fatiguable"[All Fields] OR "Fatigue"[MeSH Terms] OR "Fatigue"[All Fields] OR "fatigued"[All Fields] OR "fatigues"[All Fields] OR "fatiguing"[All Fields] OR "fatigueability"[All Fields])) AND "fibromyalgia syndrome"[Title/Abstract]) OR "chronic fatigue fibromyalgia syndromes"[Title/Abstract] OR "chronic fatigue disorder"[Title/Abstract] OR "chronic fatigue disorders"[Title/Abstract] OR "akureyri disease"[Title/Abstract] OR "benign myalgic encephalomyelitis"[Title/Abstract] OR "chronic fatigue"[Title/Abstract] OR "epidemic neuromyasthenia"[Title/Abstract] OR "fatigue syndrome"[Title/Abstract] OR "iceland disease"[Title/Abstract] OR (("syndrom"[All Fields] OR "syndromal"[All Fields] OR "syndromally"[All Fields] OR "Syndrome"[MeSH Terms] OR "Syndrome"[All Fields] OR "Syndromes"[All Fields] OR "syndrome s"[All Fields] OR "syndromic"[All Fields] OR "syndroms"[All Fields]) AND "chronic fatigue"[Title/Abstract]) OR "yuppie flu"[Title/Abstract])) AND ("corrective exercise"[Title/Abstract] OR "exercise movement techniques"[Title/Abstract] OR "exercise treatment"[Title/Abstract] OR ("kinesiotherapeutic"[All Fields] AND "intervention"[Title/Abstract]) OR ("kinesiotherapeutic"[All Fields] AND "method"[Title/Abstract]) OR ("kinesiotherapeutic"[All Fields] AND "procedure"[Title/Abstract]) OR ("kinesiotherapeutic"[All Fields] AND "technique"[Title/Abstract]) OR ("kinesiotherapeutical"[All Fields] AND "treatment"[Title/Abstract]) OR "kinesitherapeutic exercises"[Title/Abstract] OR ("kinesitherapeutic"[All Fields] AND "intervention"[Title/Abstract]) OR ("kinesitherapeutic"[All Fields] AND "method"[Title/Abstract]) OR "kinesitherapeutic methodology"[Title/Abstract] OR ("kinesitherapeutic"[All Fields] AND "procedure"[Title/Abstract]) OR ("kinesitherapeutic"[All Fields] AND "technique"[Title/Abstract]) OR "kinesitherapeutic treatment"[Title/Abstract] OR ("treatment"[Title/Abstract]) OR "kinesitherapy"[Title/Abstract] OR ("SKTM"[All Fields] AND "specialized kinesitherapeutic methodology"[Title/Abstract]) OR (("medicine"[MeSH Terms] OR "medicine"[All Fields] OR "specialty"[All Fields] OR "special"[All Fields] OR "specialisations"[All Fields] OR "specialise"[All Fields] OR "specialised"[All Fields] OR "specialises"[All Fields] OR "specialising"[All Fields] OR "specialisms"[All Fields] OR "specialities"[All Fields] OR "speciality"[All Fields] OR "specialization"[MeSH Terms] OR "specialization"[All Fields] OR "specialisation"[All Fields] OR "specialism"[All Fields] OR "specializations"[All Fields] OR "specialize"[All Fields] OR "specialized"[All Fields] OR "specializes"[All Fields] OR "specializing"[All Fields] OR "specially"[All Fields] OR "specials"[All Fields] OR "specialties"[All Fields] OR "specialty s"[All Fields]) AND "kinesitherapeutic methodology"[Title/Abstract]) OR "therapeutic exercise"[Title/Abstract]) | 2,710 | 9:26:18 |
| 2 | (((((((((((((((((((corrective exercise[Title/Abstract]) OR (exercise movement techniques[Title/Abstract])) OR (exercise treatment[Title/Abstract])) OR (kinesiotherapeutic intervention[Title/Abstract])) OR (kinesiotherapeutic method[Title/Abstract])) OR (kinesiotherapeutic procedure[Title/Abstract])) OR (kinesiotherapeutic technique[Title/Abstract])) OR (kinesiotherapeutical treatment[Title/Abstract])) OR (kinesitherapeutic exercises[Title/Abstract])) OR (kinesitherapeutic intervention[Title/Abstract])) OR (kinesitherapeutic method[Title/Abstract])) OR (kinesitherapeutic methodology[Title/Abstract])) OR (kinesitherapeutic procedure[Title/Abstract])) OR (kinesitherapeutic technique[Title/Abstract])) OR (kinesitherapeutic treatment[Title/Abstract])) OR (kinesitherapeutical treatment[Title/Abstract])) OR (kinesitherapy[Title/Abstract])) OR (SKTM (specialized kinesitherapeutic methodology[Title/Abstract]))) OR (specialised kinesitherapeutic methodology[Title/Abstract])) OR (therapeutic exercise[Title/Abstract]) |  |  | "corrective exercise"[Title/Abstract] OR "exercise movement techniques"[Title/Abstract] OR "exercise treatment"[Title/Abstract] OR ("kinesiotherapeutic"[All Fields] AND "intervention"[Title/Abstract]) OR ("kinesiotherapeutic"[All Fields] AND "method"[Title/Abstract]) OR ("kinesiotherapeutic"[All Fields] AND "procedure"[Title/Abstract]) OR ("kinesiotherapeutic"[All Fields] AND "technique"[Title/Abstract]) OR ("kinesiotherapeutical"[All Fields] AND "treatment"[Title/Abstract]) OR "kinesitherapeutic exercises"[Title/Abstract] OR ("kinesitherapeutic"[All Fields] AND "intervention"[Title/Abstract]) OR ("kinesitherapeutic"[All Fields] AND "method"[Title/Abstract]) OR "kinesitherapeutic methodology"[Title/Abstract] OR ("kinesitherapeutic"[All Fields] AND "procedure"[Title/Abstract]) OR ("kinesitherapeutic"[All Fields] AND "technique"[Title/Abstract]) OR "kinesitherapeutic treatment"[Title/Abstract] OR ("treatment"[Title/Abstract]) OR "kinesitherapy"[Title/Abstract] OR ("SKTM"[All Fields] AND "specialized kinesitherapeutic methodology"[Title/Abstract]) OR (("medicine"[MeSH Terms] OR "medicine"[All Fields] OR "specialty"[All Fields] OR "special"[All Fields] OR "specialisations"[All Fields] OR "specialise"[All Fields] OR "specialised"[All Fields] OR "specialises"[All Fields] OR "specialising"[All Fields] OR "specialisms"[All Fields] OR "specialities"[All Fields] OR "speciality"[All Fields] OR "specialization"[MeSH Terms] OR "specialization"[All Fields] OR "specialisation"[All Fields] OR "specialism"[All Fields] OR "specializations"[All Fields] OR "specialize"[All Fields] OR "specialized"[All Fields] OR "specializes"[All Fields] OR "specializing"[All Fields] OR "specially"[All Fields] OR "specials"[All Fields] OR "specialties"[All Fields] OR "specialty s"[All Fields]) AND "kinesitherapeutic methodology"[Title/Abstract]) OR "therapeutic exercise"[Title/Abstract] | 5,847,327 | 9:25:49 |
| 1 | ("Fatigue Syndrome, Chronic"[Mesh]) OR (((((((((((((((((((((Chronic Fatigue Syndromes[Title/Abstract]) OR (Chronic fatigue syndrome[Title/Abstract])) OR (Royal Free Disease[Title/Abstract])) OR (Chronic Fatigue[Title/Abstract] AND Immune Dysfunction Syndrome[Title/Abstract])) OR (Postviral Fatigue Syndrome[Title/Abstract])) OR (Postviral Fatigue Syndromes[Title/Abstract])) OR (Systemic Exertion Intolerance Disease[Title/Abstract])) OR (Myalgic Encephalomyelitis[Title/Abstract])) OR (Chronic Fatigue Syndrome[Title/Abstract])) OR (Chronic Fatigue Fibromyalgia Syndrome[Title/Abstract])) OR (Chronic Fatigue-Fibromyalgia Syndromes[Title/Abstract])) OR (Chronic Fatigue Disorder[Title/Abstract])) OR (Chronic Fatigue Disorders[Title/Abstract])) OR (Akureyri disease[Title/Abstract])) OR (benign myalgic encephalomyelitis[Title/Abstract])) OR (chronic fatigue[Title/Abstract])) OR (epidemic neuromyasthenia[Title/Abstract])) OR (fatigue syndrome[Title/Abstract])) OR (Iceland disease[Title/Abstract])) OR (syndrome of chronic fatigue[Title/Abstract])) OR (Yuppie flu[Title/Abstract])) |  |  | "fatigue syndrome, chronic"[MeSH Terms] OR ("chronic fatigue syndromes"[Title/Abstract] OR "chronic fatigue syndrome"[Title/Abstract] OR "royal free disease"[Title/Abstract] OR ("chronic fatigue"[Title/Abstract] AND "immune dysfunction syndrome"[Title/Abstract]) OR "postviral fatigue syndrome"[Title/Abstract] OR "postviral fatigue syndromes"[Title/Abstract] OR "systemic exertion intolerance disease"[Title/Abstract] OR "myalgic encephalomyelitis"[Title/Abstract] OR "chronic fatigue syndrome"[Title/Abstract] OR ((("Chronic"[All Fields] OR "chronical"[All Fields] OR "chronically"[All Fields] OR "chronicities"[All Fields] OR "chronicity"[All Fields] OR "chronicization"[All Fields] OR "chronics"[All Fields]) AND ("fatiguability"[All Fields] OR "fatiguable"[All Fields] OR "Fatigue"[MeSH Terms] OR "Fatigue"[All Fields] OR "fatigued"[All Fields] OR "fatigues"[All Fields] OR "fatiguing"[All Fields] OR "fatigueability"[All Fields])) AND "fibromyalgia syndrome"[Title/Abstract]) OR "chronic fatigue fibromyalgia syndromes"[Title/Abstract] OR "chronic fatigue disorder"[Title/Abstract] OR "chronic fatigue disorders"[Title/Abstract] OR "akureyri disease"[Title/Abstract] OR "benign myalgic encephalomyelitis"[Title/Abstract] OR "chronic fatigue"[Title/Abstract] OR "epidemic neuromyasthenia"[Title/Abstract] OR "fatigue syndrome"[Title/Abstract] OR "iceland disease"[Title/Abstract] OR (("syndrom"[All Fields] OR "syndromal"[All Fields] OR "syndromally"[All Fields] OR "Syndrome"[MeSH Terms] OR "Syndrome"[All Fields] OR "Syndromes"[All Fields] OR "syndrome s"[All Fields] OR "syndromic"[All Fields] OR "syndroms"[All Fields]) AND "chronic fatigue"[Title/Abstract]) OR "yuppie flu"[Title/Abstract]) | 11,018 | 9:25:27 |

**Attachment 6** Web of science search strategy(2025.06.07)

| Entitlements | # | Search Query | Database | Results | Date Run |  |  |  |  |
| --- | --- | --- | --- | --- | --- | --- | --- | --- | --- |
| - WOS.IC: 1993 to 2025 - WOS.CCR: 1985 to 2025 - WOS.SCI: 2000 to 2025 - WOS.AHCI: 2000 to 2025 - WOS.ESCI: 2020 to 2025 - WOS.ISTP: 2006 to 2025 - WOS.SSCI: 2000 to 2025 - WOS.ISSHP: 2006 to 2025 | 11 | ((((((((((((((((((((TS=(Chronic fatigue syndrome)) OR TS=(Chronic Fatigue Syndromes))) OR TS=(Royal Free Disease)) OR TS=(Chronic Fatigue and Immune Dysfunction Syndrome)) OR TS=(Postviral Fatigue Syndrome)) OR TS=(Postviral Fatigue Syndromes)) OR TS=(Systemic Exertion Intolerance Disease)) OR TS=(Chronic Fatigue Syndrome)) OR TS=(Chronic Fatigue Fibromyalgia Syndrome)) OR TS=(Chronic Fatigue-Fibromyalgia Syndromes)) OR TS=(Chronic Fatigue Disorder)) OR TS=(Chronic Fatigue Disorders)) OR TS=(Akureyri disease)) OR TS=(benign myalgic encephalomyelitis)) OR TS=(chronic fatigue)) OR TS=(epidemic neuromyasthenia)) OR TS=(fatigue syndrome)) OR TS=(Iceland disease)) OR TS=(syndrome of chronic fatigue )) OR TS=(Yuppie flu) | Web of Science Core Collection | 36623 | Sat Jun 07 2025 21:45:32 GMT+0800 |  |  |  |  |
| - WOS.IC: 1993 to 2025 - WOS.CCR: 1985 to 2025 - WOS.SCI: 2000 to 2025 - WOS.AHCI: 2000 to 2025 - WOS.ESCI: 2020 to 2025 - WOS.ISTP: 2006 to 2025 - WOS.SSCI: 2000 to 2025 - WOS.ISSHP: 2006 to 2025 | 12 | (((((((((((((((((((((TS=(Exercise therapy)) OR TS=(kinesiotherapy)) OR TS=(corrective exercise)) OR TS=(exercise movement techniques)) OR TS=(exercise treatment)) OR TS=(kinesiotherapeutic intervention)) OR TS=(kinesiotherapeutic method)) OR TS=(kinesiotherapeutic procedure)) OR TS=(kinesiotherapeutic technique)) OR TS=(kinesiotherapeutical treatment)) OR TS=(kinesitherapeutic exercises)) OR TS=(kinesitherapeutic intervention)) OR TS=(kinesitherapeutic method)) OR TS=(kinesitherapeutic methodology)) OR TS=(kinesitherapeutic procedure)) OR TS=(kinesitherapeutic technique)) OR TS=(kinesitherapeutic treatment)) OR TS=(kinesitherapeutical treatment)) OR TS=(kinesitherapy)) OR TS=(SKTM (specialized kinesitherapeutic methodology))) OR TS=(specialised kinesitherapeutic methodology)) OR TS=(therapeutic exercise) | Web of Science Core Collection | 124191 | Sat Jun 07 2025 21:45:53 GMT+0800 |  |  |  |  |
| - WOS.IC: 1993 to 2025 - WOS.CCR: 1985 to 2025 - WOS.SCI: 2000 to 2025 - WOS.AHCI: 2000 to 2025 - WOS.ESCI: 2020 to 2025 - WOS.ISTP: 2006 to 2025 - WOS.SSCI: 2000 to 2025 - WOS.ISSHP: 2006 to 2025 | 13 | #11 AND #12 | Web of Science Core Collection | 2517 | Sat Jun 07 2025 21:46:08 GMT+0800 |  |  |  |  |
| - WOS.IC: 1993 to 2025 - WOS.CCR: 1985 to 2025 - WOS.SCI: 2000 to 2025 - WOS.AHCI: 2000 to 2025 - WOS.ESCI: 2020 to 2025 - WOS.ISTP: 2006 to 2025 - WOS.SSCI: 2000 to 2025 - WOS.ISSHP: 2006 to 2025 | 14 | DOP=(2024-04-01/2025-06-07) | Web of Science Core Collection | 3642396 | Sat Jun 07 2025 21:47:25 GMT+0800 (中国标准时间) |  |  |  |  |
| - WOS.IC: 1993 to 2025 - WOS.CCR: 1985 to 2025 - WOS.SCI: 2000 to 2025 - WOS.AHCI: 2000 to 2025 - WOS.ESCI: 2020 to 2025 - WOS.ISTP: 2006 to 2025 - WOS.SSCI: 2000 to 2025 - WOS.ISSHP: 2006 to 2025 | 15 | #13 AND #14 | Web of Science Core Collection | 229 | Sat Jun 07 2025 21:47:36 GMT+0800 (中国标准时间) |  |  |  |  |

**Attachment 7** Embase(2025.06.07)

| No. | Query Results | Results | Date |
| --- | --- | --- | --- |
| #1. | ('chronic fatigue syndrome'/exp OR 'chronic fatigue syndrome') AND [2024-2025]/py AND [01-04-2024]/sd | 4,447 | 7 Jun 2025 |
| #2. | ('chronic fatigue syndromes':ab,ti OR 'royal free  disease':ab,ti OR ('chronic fatigue':ab,ti AND  'immune dysfunction syndrome':ab,ti) OR  'postviral fatigue syndrome':ab,ti OR 'postviral  fatigue syndromes':ab,ti OR 'systemic exertion  intolerance disease':ab,ti OR 'myalgic  encephalomyelitis':ab,ti OR 'chronic fatigue  syndrome':ab,ti OR 'chronic fatigue fibromyalgia  syndrome':ab,ti OR 'chronic fatigue-fibromyalgia  syndromes':ab,ti OR 'chronic fatigue  disorder':ab,ti OR 'chronic fatigue  disorders':ab,ti OR 'akureyri disease':ab,ti OR  'benign myalgic encephalomyelitis':ab,ti OR  'chronic fatigue':ab,ti OR 'epidemic  neuromyasthenia':ab,ti OR 'fatigue  syndrome':ab,ti OR 'iceland disease':ab,ti OR  'syndrome of chronic fatigue':ab,ti OR 'yuppie  flu':ab,ti) AND [2024-2025]/py AND  [01-04-2024]/sd | 770 | 7 Jun 2025 |
| #3. | #1 OR #2 | 4,516 | 7 Jun 2025 |
| #4. | ('kinesiotherapy'/exp OR 'kinesiotherapy') AND  [2024-2025]/py AND [01-04-2024]/sd | 9,812 | 7 Jun 2025 |
| #5. | ('corrective exercise':ab,ti OR 'exercise  movement techniques':ab,ti OR 'exercise  treatment':ab,ti OR 'kinesiotherapeutic  intervention':ab,ti OR 'kinesiotherapeutic  method':ab,ti OR 'kinesiotherapeutic  technique':ab,ti OR 'kinesiotherapeutic  procedure':ab,ti OR 'kinesiotherapeutical  treatment':ab,ti OR 'kinesitherapeutic  exercises':ab,ti OR 'kinesitherapeutic  intervention':ab,ti OR 'kinesitherapeutic  method':ab,ti OR 'kinesitherapeutic  methodology':ab,ti OR 'kinesitherapeutic  procedure':ab,ti OR 'kinesitherapeutic  technique':ab,ti OR 'kinesitherapeutic  treatment':ab,ti OR 'kinesitherapeutical  treatment':ab,ti OR kinesitherapy:ab,ti OR  (sktm:ab,ti AND 'specialized kinesitherapeutic  methodology':ab,ti) OR 'specialised  kinesitherapeutic methodology':ab,ti OR  'specialized kinesitherapeutic methodology':ab,ti  OR 'therapeutic exercise':ab,ti) AND  [2024-2025]/py AND [01-04-2024]/sd | 363 | 7 Jun 2025 |
| #6. | #4 OR #5 | 9,870 | 7 Jun 2025 |
| #7. | #3 AND #6 | 119 | 7 Jun 2025 |

**Attachment 8** Chocrane(2025.06.07)

Date Run: 07/06/2025 21:27:22

Comment:

| ID | Search | Hits |
| --- | --- | --- |
| #1 | MeSH descriptor: [Fatigue Syndrome, Chronic] explode all trees | 565 |
| #2 | (Chronic Fatigue Syndromes):ti,ab,kw OR (Royal Free Disease):ti,ab,kw OR (Chronic Fatigue and Immune Dysfunction Syndrome):ti,ab,kw OR (Postviral Fatigue Syndrome):ti,ab,kw OR (Postviral Fatigue Syndromes):ti,ab,kw (Word variations have been searched) | 474 |
| #3 | (Systemic Exertion Intolerance Disease):ti,ab,kw OR (Myalgic Encephalomyelitis):ti,ab,kw OR (Chronic Fatigue Syndrome):ti,ab,kw OR (Chronic Fatigue Fibromyalgia Syndrome):ti,ab,kw OR (Chronic Fatigue-Fibromyalgia Syndromes):ti,ab,kw (Word variations have been searched) | 2381 |
| #4 | (Chronic Fatigue Disorder):ti,ab,kw OR (Chronic Fatigue Disorders):ti,ab,kw OR (Akureyri disease):ti,ab,kw OR (benign myalgic encephalomyelitis):ti,ab,kw OR (chronic fatigue):ti,ab,kw (Word variations have been searched) | 9199 |
| #5 | (epidemic neuromyasthenia):ti,ab,kw OR (fatigue syndrome):ti,ab,kw OR (Iceland disease):ti,ab,kw OR (syndrome of chronic fatigue):ti,ab,kw OR (Yuppie flu):ti,ab,kw (Word variations have been searched) | 6847 |
| #6 | #1 OR #2 OR #3 OR #4 OR #5 | 13779 |
| #7 | MeSH descriptor: [Exercise Therapy] explode all trees | 22846 |
| #8 | (kinesiotherapy):ti,ab,kw OR (corrective exercise):ti,ab,kw OR (exercise movement techniques):ti,ab,kw OR (exercise treatment):ti,ab,kw OR (kinesiotherapeutic intervention):ti,ab,kw (Word variations have been searched) | 55066 |
| #9 | (kinesiotherapeutic method):ti,ab,kw OR (kinesiotherapeutic procedure):ti,ab,kw OR (kinesiotherapeutic technique):ti,ab,kw OR (kinesiotherapeutical treatment):ti,ab,kw OR (kinesitherapeutic exercises):ti,ab,kw (Word variations have been searched) | 3 |
| #10 | (kinesitherapeutic intervention):ti,ab,kw OR (kinesitherapeutic methodology):ti,ab,kw OR (kinesitherapeutic method):ti,ab,kw OR (kinesitherapeutic procedure):ti,ab,kw OR (kinesitherapeutic technique):ti,ab,kw (Word variations have been searched) | 5 |
| #11 | (kinesitherapeutic treatment):ti,ab,kw OR (kinesitherapeutical treatment):ti,ab,kw OR (kinesitherapy):ti,ab,kw OR (SKTM (specialized kinesitherapeutic methodology)):ti,ab,kw OR (specialised kinesitherapeutic methodology):ti,ab,kw (Word variations have been searched) | 122 |
| #12 | (specialized kinesitherapeutic methodology):ti,ab,kw OR (therapeutic exercise):ti,ab,kw (Word variations have been searched) | 15949 |
| #13 | #7 OR #8 OR #9 OR #10 OR #11 OR #12 | 71474 |
| #14 | #6 AND #13 with Cochrane Library publication date Between Apr 2024 and Jun 2025 | 203 |
